# A Nanoencapsulated Ir(III)-Phthalocyanine Conjugate as a Promising Photodynamic Therapy Anticancer Agent

**DOI:** 10.1021/acsami.4c05181

**Published:** 2024-07-23

**Authors:** Joaquín Bonelli, Enrique Ortega-Forte, Gloria Vigueras, Jorge Follana-Berná, Pezhman Ashoo, Diego Abad-Montero, Neus Isidro, Marta López-Corrales, Adrián Hernández, Javier Ortiz, Eduardo Izquierdo-García, Manel Bosch, Josep Rocas, Ángela Sastre-Santos, José Ruiz, Vicente Marchán

**Affiliations:** † Departament de Química Inorgànica i Orgànica, Secció de Química Orgànica, Universitat de Barcelona (UB), and Institut de Biomedicina de la Universitat de Barcelona (IBUB), Martí i Franquès 1-11, E-08028 Barcelona, Spain; ‡ Ecopol Tech S.L., Nanobiotechnological Polymers Division, R&D Department, El Foix Business Park, Indústria 7, E-43720 L’Arboç del Penedès, Tarragona, Spain; § Departamento de Química Inorgánica, Universidad de Murcia, and Institute for Bio-Health Research of Murcia (IMIB-Arrixaca), E-30100 Murcia, Spain; ∥ Área de Química Orgánica, Instituto de Bioingeniería, Universidad Miguel Hernández, Avda. de la Universidad s/n, E-03203 Elche, Spain; ⊥ Unitat de Microscòpia Òptica Avançada, Centres Científics i Tecnològics, Universitat de Barcelona, Av. Diagonal 643, E-08028 Barcelona, Spain

**Keywords:** zinc phthalocyanines, cyclometalated iridium(III) complexes, photodynamic therapy, anticancer agents, drug design, photosensitizer, hypoxia, nanoencapsulation

## Abstract

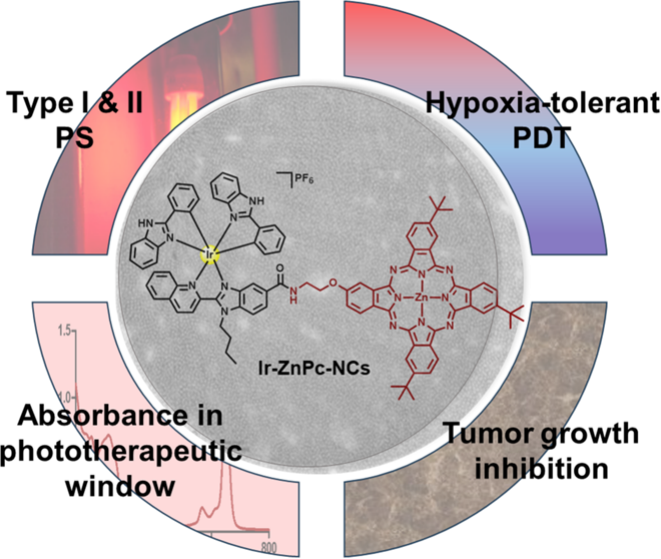

Despite the potential of photodynamic therapy (PDT) in cancer treatment, the development of efficient and photostable photosensitizing molecules that operate at long wavelengths of light has become a major hurdle. Here, we report for the first time an Ir(III)-phthalocyanine conjugate (**Ir-ZnPc**) as a novel photosensitizer for high-efficiency synergistic PDT treatment that takes advantage of the long-wavelength excitation and near infrared (NIR) emission of the phthalocyanine scaffold and the known photostability and high phototoxicity of cyclometalated Ir(III) complexes. In order to increase water solubility and cell membrane permeability, the conjugate and parent zinc phthalocyanine (**ZnPc**) were encapsulated in amphoteric redox-responsive polyurethane-polyurea hybrid nanocapsules (**Ir-ZnPc-NCs** and **ZnPc-NCs**, respectively). Photobiological evaluations revealed that the encapsulated **Ir-ZnPc** conjugate achieved high photocytotoxicity in both normoxic and hypoxic conditions under 630 nm light irradiation, which can be attributed to dual Type I and Type II reactive oxygen species (ROS) photogeneration. Interestingly, PDT treatments with **Ir-ZnPc-NCs** and **ZnPc-NCs** significantly inhibited the growth of three-dimensional (3D) multicellular tumor spheroids. Overall, the nanoencapsulation of Zn phthalocyanines conjugated to cyclometalated Ir(III) complexes provides a new strategy for obtaining photostable and biocompatible red-light-activated nano-PDT agents with efficient performance under challenging hypoxic environments, thus offering new therapeutic opportunities for cancer treatment.

## Introduction

Photodynamic therapy (PDT) represents an excellent strategy for treating cancer, which is still one of the most important health problems worldwide.^1^ In PDT, tumor cell death is induced by the combined effect of three components when overlapped spatiotemporally at the tumor place: a photosensitizer drug (PS), oxygen and light of a suitable wavelength, which results in the formation of highly cytotoxic reactive oxygen species (ROS).^1^ Upon light irradiation, the PS is activated to the nanosecond-lived excited singlet state, which quickly converts to a more stable excited triplet state via intersystem crossing. This triplet state PS exists long enough to trigger the generation of several ROS such as superoxide (^•^O_2_^–^), hydroxyl radical (^•^OH), hydrogen peroxide (H_2_O_2_), and peroxyl radicals (ROO^•^) through an electron transfer mechanism (Type-I PDT) and/or of singlet oxygen (^1^O_2_) through energy transfer to ground-state triplet oxygen (^3^O_2_) (Type-II PDT), which cause damage to the tumor cells and vasculatures by inducing different cell-death mechanisms (e.g., apoptosis and/or necrosis) or by activating the immune response.^2,3^ However, one of the salient features of solid tumors is hypoxia, which also associates with poor prognosis for cancer patients.^4^ The fact that the mechanism of action of most clinically approved PSs relies on the generation of singlet oxygen, which depends exclusively on oxygen concentration, limits the treatment of hypoxic solid tumors.^5−8^ PSs that are activatable with light irradiation within the phototherapeutic window (650–900 nm), where the endogenous chromophores of the human body do not absorb, would achieve great tissue penetration. Therefore, the development of novel PSs with operability under hypoxia and in the phototherapeutic window is key to facilitate the treatment of deep-seated hypoxic tumors with PDT.^9,10^

Phthalocyanines (Pcs) have been investigated as PSs for PDT since they exhibit optimal light absorption in the range from 650 to 850 nm with high extinction coefficients.^11,12^ On the contrary, Pcs show low or no absorption at 400–600 nm, which is highly desirable to minimize skin phototoxicity.^13^ Furthermore, Pcs are able to coordinate more than 70 metallic elements in their central cavity and can be functionalized in the axial, peripheral, and nonperipheral positions, which allows tuning their photophysical and chemical properties.^14^ Due to the potential of Pcs in PDT, some of them have entered clinical trials for the treatment of different cancers in some countries (e.g., Photosens in Russia and Photocyanine in China).^15,16^ Despite these advantages, Pcs can produce toxicity when exposed to light due to the formation of photodegradation products and show strong aggregation in aqueous solution that hamper cellular uptake and produce fluorescence quenching and ROS generation reduction.^17^ Functionalization of the axial positions of Pcs with sulfonic groups as well as liposomal formulations have been explored as possible solutions to improve biocompatibility by increasing water solubility.^12,18^ In addition, tumor selectivity can be achieved through the incorporation of targeting moieties within the Pc scaffold.^19^

**Figure 1:**
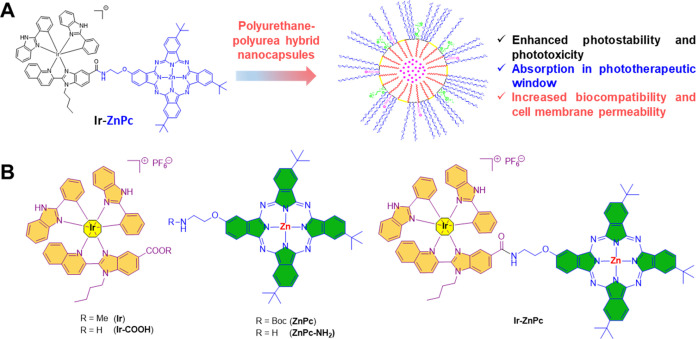
(A) Strategy overview of the current work in which we have conjugated a zinc phthalocyanine (**ZnPc**) which exhibits excellent absorption into the phototherapeutic window to a highly photostable and phototoxic cyclometalated Ir(III) complex. The conjugate has been encapsulated in amphoteric redox-responsive polyurethane-polyurea hybrid nanocapsules in order to increase the water solubility and cell membrane permeability. (B) Structure of zinc phthalocyanines and cyclometalated Ir(III) complexes used in this work, and of the corresponding Ir(III)-phthalocyanine conjugate (**Ir-ZnPc**).

Transition metal complexes have also been positioned in recent years as promising PSs for anticancer PDT applications, particularly those based on cyclometalated Ir(III) complexes and on Ru(II) polypyridyl complexes.^20−26^ Nevertheless, metal complexes frequently suffer from high dark cytotoxicity, strong dependence on oxygen supply, and absorption outside the phototherapeutic window, which is difficult for clinical translation.^9^ A promising approach to tackle these limitations consists of combining them with suitable organic fluorophores that absorb in the far-red/near-infrared (NIR) region of the electromagnetic spectrum, such as BODIPY or coumarin scaffolds. In this context, we have recently described a novel class of PSs based on the conjugation of a cyclometalated Ir(III) complex (**Ir**, Figure 1) to far-red emitting COUPY coumarin fluorophores^27,28^ exhibiting high photoactivities upon visible light irradiation under hypoxic conditions, which is a consequence of the selective generation of superoxide anion radicals.^29−31^ Similarly, the conjugation of a cyclometalated Ru(II) polypyridyl complex to a COUPY coumarin provided a PS that was also found to be highly phototoxic under hypoxia.^32^ Based on these antecedents, we envisaged Ir(III)-phthalocyanine conjugates as novel PSs for high-efficiency synergistic PDT treatment^11,33^ taking advantage of the long-wavelength excitation and NIR emission of the phthalocyanine scaffold and of the known phototoxicities of transition metal complexes, in this case of a cyclometalated Ir(III) complex. Ultimately, we predicted that nanoencapsulation of such conjugates would allow efficient cell delivery and avoid solubility issues commonly associated with Pc-based compounds. In this context, polyurethane-polyurea hybrid nanocapsules (NCs) based on ECOSTRATAR technology^34^ have been shown to successfully enhance long circulation, cell penetration, and biocompatibility of hydrophobic compounds,^35,36^ including poorly water-soluble neutral tris-cyclometalated Ir(III) anticancer complexes,^37^ and to improve the anticancer phototherapeutic profile of coumarin-based PSs.^38,39^

Armed with learnings from these previous studies, herein, we report for the first time the development of a PS based on the conjugation of a zinc phthalocyanine (**ZnPc**) to a cyclometataled Ir(III) complex (**Ir-ZnPc**, Figure 1), and its encapsulation into amphoteric redox-responsive polyurethane-polyurea hybrid NCs. The present study explored the potential of these nanoformulations as PDT anticancer agents under challenging hypoxic conditions.

## Results and Discussion

### Synthesis and Characterization of Ir(III)-Phthalocyanine Conjugate and of ZnPc- and Ir-ZnPc-Loaded NCs

**Figure 2:**
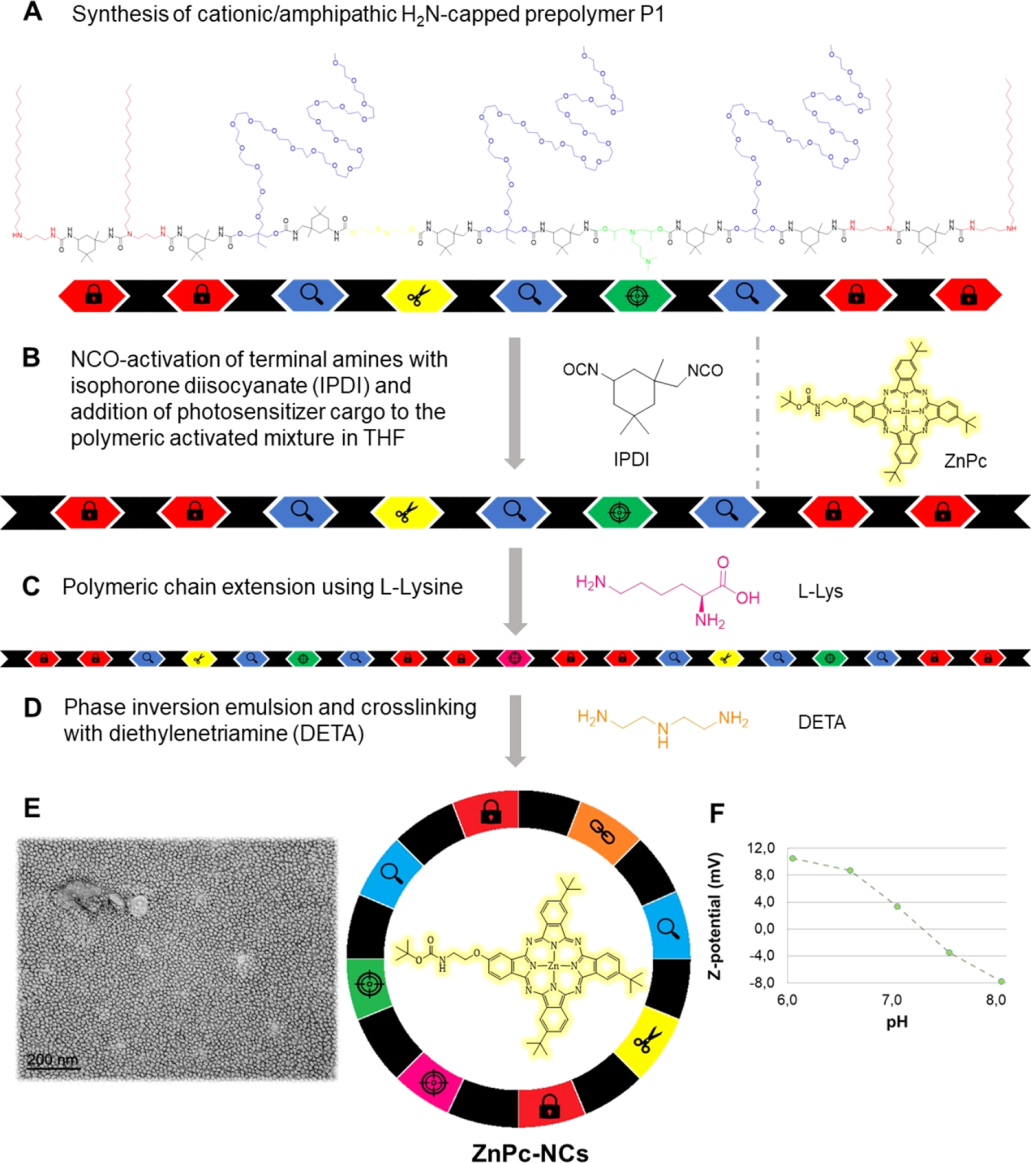
(A–D) Schematic representation of the synthesis of **ZnPc**-loaded amphoteric redox-responsive polyurethane/polyurea hybrid nanocapsules. (E) TEM micrograph of **ZnPc-NCs**. (F) ζ-potential values of **ZnPc**-loaded NCs vs pH.

The synthesis of the Ir(III)-phthalocyanine conjugate (**Ir-ZnPc**) was carried out through the formation of an amide bond between the amino group of **ZnPc-NH**_**2**_(40) and the corresponding Ir(III) complex bearing a carboxyl group (**Ir-COOH**).^29^ In order to prevent Pc aggregation, *tert*-butyl groups were introduced at the Pc macrocycle nonperipheral position. **Ir-ZnPc** conjugate was obtained with excellent yield (91%) as a dark blue solid after purification by silica column chromatography and fully characterized by NMR spectroscopy (Figures S1 and S2) and high resolution-matrix-assisted laser desorption ionization-time-of-flight (HR-MALDI-TOF) mass spectrometry (MS) (Figure S3). On the other hand, the corresponding nanoformulations, **ZnPc-NCs** and **Ir-ZnPc-NCs**, were satisfactorily synthesized following the methodology previously used for the encapsulation of liposoluble compounds based on amphoteric redox-responsive polyurethane-polyurea hybrid nanocapsules (Figure 2). In the first step, a cationic redox-responsive polyurethane polymer (P1) which laterally includes PEG1000, tertiary diamines, and linear disulfide hanging groups was synthesized and subsequently capped, through polyurea formation, with hydrophobic diamino groups in THF. For the synthesis of the nanocapsules, the photosensitizer (**Zn-Pc** or **Ir-ZnPc**) was first solubilized in a mixture of caprylic/capric triglyceride (GTCC) and the NH_2_-reactive P1 in THF. Afterward, the activation of the amino groups of P1 was carried out with isophorone diisocyanate (IPDI), which allowed their subsequent polymer chain extension using l-lysine. Once the lysine groups had been introduced, the polymeric backbone was emulsified in aqueous media to be then cross-linked, in a final step, using diethylenetriamine (DETA), furnishing the nanocapsules’ wall. This synthetic methodology was carried out in a one-pot process without the use of any external emulsifiers, requiring a dialysis purification to remove overage polymeric moieties and the nonencapsulated part of the active cargo. After dialysis purification, the quantification of zinc and iridium content in the nanocapsules’ emulsions by inductively coupled plasma-MS (ICP-MS) allowed us to determine both the cargo concentration and the encapsulation efficacy of the methodology for these compounds. In both cases, the encapsulation efficiency, 64% for **ZnPc-NCs** and 50% for **Ir-ZnPc-NCs**, were in good agreement with the high lipophilicity of the compounds. The dynamic light scattering (DLS) values for **ZnPc**- and **Ir-ZnPc**-loaded nanocapsules (Table S3 and Figures S6 and S7) are in good agreement with the expected values for the ratio of dispersible phase and self-emulsifiable polymer. Using similar polymer/hydrophobic-phase ratios for other anticancer agents encapsulated using polyurethane-polyurea hybrid nanocapsules, slight but not significant differences were observed in terms of size. The molecular weight, the aggregation events, and especially the intrinsic hydrophobicity of the encapsulated molecules have been defined as crucial parameters for a proper encapsulation in a liposoluble core of polyurethane-polyurea hybrid nanocapsules. The spherical morphology and diameter of dried **ZnPc-NCs** and **Ir-ZnPc-NCs** were also confirmed by transmission electron microscopy (TEM) analyses (Figures 2 and S8). As indicated in Table S4 and Figures 2, S9, and S10, the surface charge of **ZnPc**- and **Ir-ZnPc**-loaded nanocapsules was dependent on the pH of the media. These results were expected and parallel to those obtained before with COUPY- and Ir(III)-loaded nanocapsules.^37,38^ Indeed, amphoteric functionalization of the nanocapsules’ surface allows protonation under the acidic pH found in the tumor microenvironment. As observed in other NCs prepared by using the same system, ζ-potential values are only related to the degree of ionomeric moieties incorporated at the surface of the nanocapsules, masking the charge that could be provided by the encapsulated molecule.

### Photophysical and Photochemical Characterization

The photophysical properties of the conjugate (**Ir–ZnPc**) were first studied in CH_2_Cl_2_ and dimethyl sulfoxide (DMSO) and compared with those of the parent Ir(III) complex (**Ir**) and Zn phthalocyanine (**ZnPc**) (Figure S11 and Table 1). The ultraviolet–visible (UV–vis) absorption spectra of the **Ir–ZnPc** conjugate in the visible region are dominated by strong bands around 600–700 nm originating from the **ZnPc** fragment (Q-band), and consequently, the wavelength and molar absorptivity (ε) of such visible light absorption bands are identical to those of the unconjugated zinc phthalocyanine. In contrast, in the UV region, the molar absorptivity was found slightly larger for the conjugate than for the free phthalocyanine due to the contribution of the cyclometalated Ir(III) complex. The emission of the **Ir-ZnPc** conjugate was identical to that of the free **ZnPc** compound in both solvents, whereas the Ir(III) complex did not show any luminescence upon red light excitation since it does not absorb in this region of the electromagnetic spectrum. At 370 nm excitation, where both the iridium fragment and the Zn phthalocyanine absorb, the conjugate showed only one band at 688 nm due to the **ZnPc** fragment. In contrast, a broad band at 670 nm was observed for the free Ir(III) complex (Figures S12 and S13). The emission lifetimes of the **Ir–ZnPc** conjugate (Table 1) were quite similar to those of the free **ZnPc** in both solvents, showing a biexponential decay with a short (222–247 ns) and a very long (1210–1340 ns) component. On the other hand, emission quantum yields were significantly lower for the conjugate compared with the **ZnPc** (Φ_DMSO_ = 0.07 and 0.20, respectively), probably due to the existence of competitive excited-state processes.^29,41^

**Table 1:** Absorption (λ_abs_) and Emission (λ_em_) Maxima Wavelengths, Emission Lifetimes (τ_em_), and Quantum Yields (Φ_em_) of the Iridium Complex (**Ir**), Zn Phthalocyanine (**ZnPc**), and the Conjugate (**Ir-ZnPc**) in DMSO and CH_2_Cl_2_, and of the Corresponding Nanoformulations (**ZnPc-NCs** and **Ir-ZnPc-NCs**) in Water

				degassed	
compound	solvent	λ_abs_, nm	λ_em_, nm	τ_em_, ns^a^	Φ_em_^a^
**Ir**	CH_2_Cl_2_	360	670	62.7 (12%), 30.5 (88%)	0.058
	DMSO	370	670	13.0 (7%), 31.5 (93%)	0.022
**ZnPc**	CH_2_Cl_2_	676	686	235 (25%), 1259 (75%)	0.283
	DMSO	678	688	247 (24%), 1329 (76%)	0.207
**Ir-ZnPc**	CH_2_Cl_2_	677	686	227 (22%), 1210 (78%)	0.184
	DMSO	679	688	222 (20%), 1340 (80%)	0.070
**ZnPc-NCs**	water	678	689	211 (23%), 1190 (77%)	0.053
**Ir-ZnPc-NCs**	water	679	688	89.4 (7%) 322 (45%), 1130 (48%)	0.201

a Deaerated solution 20 min under argon.

**Figure 3:**
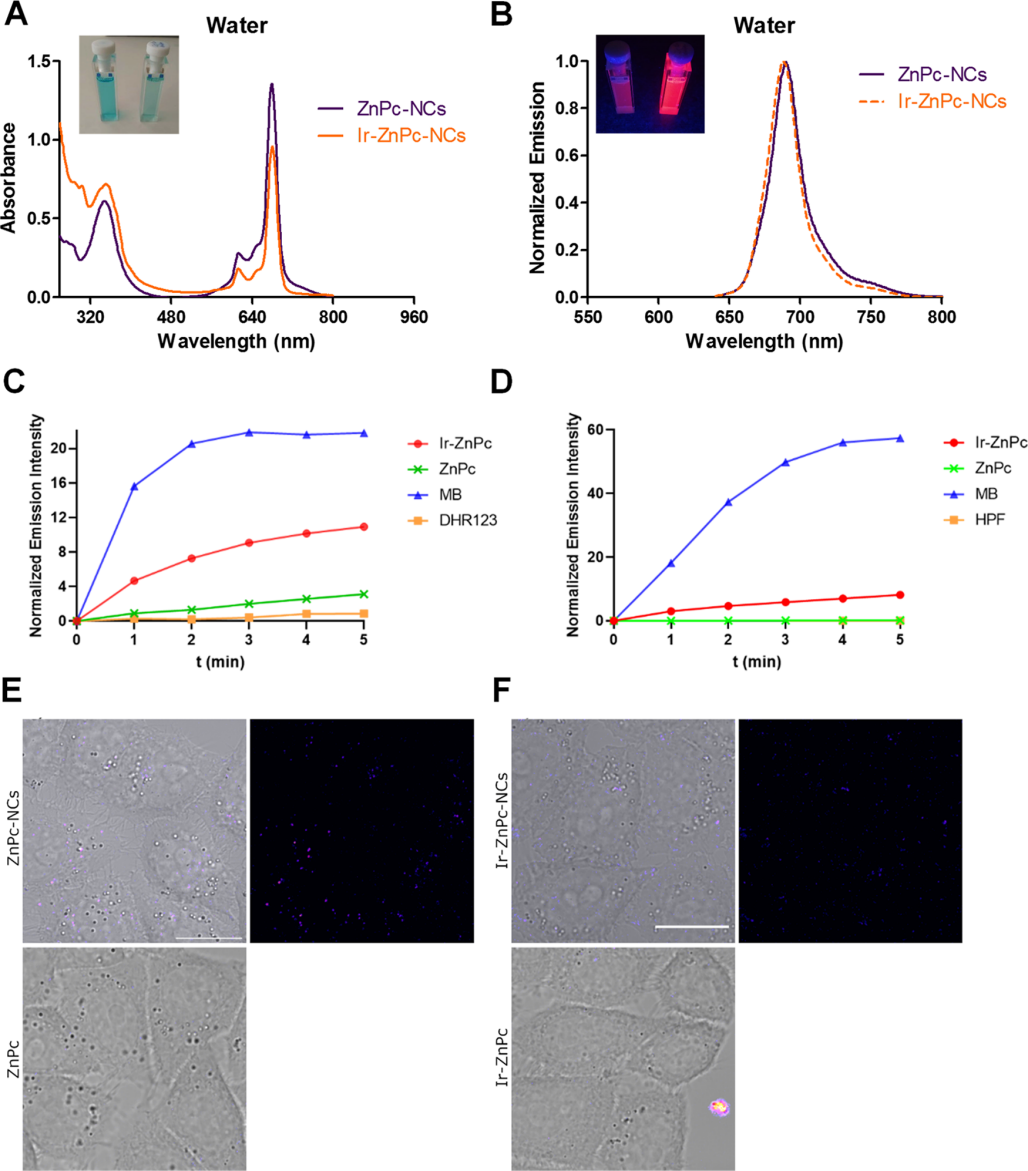
UV–visible (A) and normalized emission spectra (B) of **ZnPc**- and **Ir-ZnPc**-loaded NCs in H_2_O (λ_exc_ = 620 nm). Inset: Photographic images of NCs in daylight (left) and in the dark (right) upon irradiation with a blue light laser (465 nm). (C, D) Photogeneration of superoxide and hydroxyl radical by **ZnPc** and **Ir-ZnPc**. Increase of the fluorescence spectra emission of DHR123 (C) or HPF (D) upon photoirradiation of **ZnPc**, **Ir-ZnPc**, and methylene blue (**MB**) or without any compound (DHR123 or HPF alone) at 620 nm (130 mW/cm^2^) in PBS (0.2% DMSO). DHR123 and HPF fluorescence were excited at 500 and 490 nm, respectively. (E, F) Single confocal planes of HeLa cells incubated with the encapsulated and free forms of the compounds for 30 min at 37 °C. In (E, F), left-hand side shows the merge of bright-field and fluorescence images and right-hand side shows the fluorescence images of the compounds. (E) **ZnPc-NCs** (top) and **ZnPc** (bottom). (F) **Ir-ZnPc-NCs** (top) and **Ir-ZnPc** (bottom). Images were acquired by irradiation with a 633 nm laser line. Scale bar: 20 μm. Adapted from ref (39)

The effect of encapsulation on the spectroscopic and photophysical properties of the **ZnPc** and the **Ir-ZnPc** conjugate (absorption and emission spectra (Figure 3A,B), as well as lifetimes (τ_em_) and emission quantum yields (Φ)) was also investigated. As shown in Figure 3A, aqueous solutions of **ZnPc** and **Ir–ZnPc** nanocapsules showed a cyan color, owing to the intense absorption band in the red region of the electromagnetic spectrum with an absorption maximum centered at ∼678 nm. Interestingly, the absorption maximum of the **Ir–ZnPc-NCs** in water was similar to that of the nonencapsulated conjugate in both organic solvents (Table 1). This fact accounts for the hydrophobic and protective environments inside the nanocapsules. After irradiation at the maximum absorption wavelength, similar emission bands were also obtained both for the encapsulated (λ_em_ = 688–689 nm) and free compounds (λ_em_ = 686–688 nm). The fact that no significant differences between the UV–visible spectra of free and encapsulated **ZnPc** and **Ir-ZnPc** compounds were observed indicates that the encapsulation process in polyurethane-polyurea hybrid nanocapsules does not compromise the chemical integrity of the photosensitizers. On the other hand, the luminescence lifetime of **ZnPc-NCs** was very similar to that of the free **ZnPc** in CH_2_Cl_2_, which again is indicative of the hydrophobicity generated by the nanoparticles. Similar luminescence quantum yields were also obtained for the **Ir-ZnPc** conjugate, either free (Φ = 0.18 in CH_2_Cl_2_) or encapsulated (Φ = 0.20 in H_2_O).

The photostability of the investigated compounds was studied by UV-vis spectroscopy after irradiation with red light (λ = 630 nm, 89 mW/cm^2^) for 1 h. As observed in Figures S15–S17, both iridium conjugation and nanoencapsulation had a clear positive effect on the photostability of Zn phthalocyanine. Indeed, as shown in Figure S17, the decrease of the absorbance of the **Ir-ZnPc** conjugate at the absorption maximum was much smaller when encapsulated in the hydrophobic environment of the polyurethane-polyurea hybrid NCs, which is in good agreement with previous results with both organic and metal-based PSs.^37,38^

Furthermore, the singlet oxygen generation by **ZnPc** and **Ir-ZnPc** conjugate, either free or nanoencapsulated, was studied by using 1,3-diphenylisobenzofuran (DPBF) as a ^1^O_2_ scavenger and methylene blue (MB) as a reference under red light irradiation (620 nm, 130 mW/cm^2^). MB is widely recognized for its ability to produce singlet oxygen and is commonly used to determine Type II PDT efficiency, although it can also produce Type I ROS such as superoxide and hydroxyl radical (see below).^42^ In all cases, the absorbance of DPBF at 411 nm was decreased in the presence of the compounds, which confirmed the generation of singlet oxygen (Figures S18 and S19), resulting in high singlet oxygen quantum yields (Φ_Δ_ = 0.54–0.65; Table S5). Interestingly, conjugation of the Ir(III) complex to the zinc phthalocyanine led to a slight increase in the singlet oxygen generation (Φ_Δ_ = 0.61 for **ZnPc** vs Φ_Δ_ = 0.65 for **Ir-ZnPc**), and nanoencapsulation did not significantly affect these values (Φ_Δ_ = 0.54 for **ZnPc** vs Φ_Δ_ = 0.58 for **Ir-ZnPc**).

Since one of the main advantages of Ir(III)-COUPY and Ru(II)-COUPY conjugates for anticancer PDT applications is the photogeneration of superoxide (^•^O_2_^–^) in living cells,^31,32^ we investigated the ability of **ZnPc** and **Ir-ZnPc** to produce this Type-I ROS by using a spectroscopic method based on dihydrorhodamine 123 (DHR123) probe. As shown in Figures 3C and S21, conjugation of the Ir(III) complex to the Zn phthalocyanine has a clear effect on the generation of superoxide upon red light irradiation, which reproduces the behavior previously found when this metal complex was conjugated to COUPY coumarins.^29^ Based on these results, we investigated whether **Ir-ZnPc** could also photogenerate hydroxyl radical (^•^OH) by using a hydroxyphenyl fluorescein (HPF) probe (Figures 3D and S22). Interestingly, **ZnPc** did not produce any measurable quantity of hydroxyl radical upon irradiation, whereas **Ir-ZnPc** clearly increased the fluorescence intensity of HPF, although to a much lesser extent compared to that of the control methylene blue. Thus, besides superoxide, the **Ir-ZnPc** conjugate can photogenerate other Type-I ROS such as hydroxyl radical.

### Cellular Uptake by Confocal Microscopy

The cellular uptake of **ZnPc** and **Ir-ZnPc** as well as of the polyurethane-polyurea NCs’ formulations was investigated by confocal microscopy by taking advantage of the luminescent properties of the zinc phthalocyanine scaffold. As shown in Figure 3E,F, confocal microscopy studies with **ZnPc-NCs** and **Ir-ZnPc-NCs** confirmed the internalization of the encapsulated compounds after incubation for 30 min in HeLa cells, suggesting a vesicular intracellular distribution pattern. By contrast, the nonencapsulated compounds remained within the extracellular media, adhered to the outer part of the cellular membrane forming aggregates, although a major part of them was removed after washing cycles. The lack of internalization of **ZnPc** and its Ir(III) conjugate could be attributed to the poor aqueous solubility of the compounds, which caused precipitation in the biological media of cell cultures.

Lipophilicity is a physicochemical parameter that strongly influences both the cellular uptake and subcellular localization of a molecule.^43^ Accordingly, we determined the distribution coefficients between octanol and water (log* P*_O/W_) of **ZnPc** and **Ir-ZnPc** as well as of **Ir** (Figure S24). All three compounds were mainly found in the octanol phase and their log *P*_O/W_ values followed the order **Ir** (2.07) < **ZnPc** (3.57) < **Ir-ZnPc** (4.00), being **ZnPc** and **Ir-ZnPc** the most lipophilic compounds, which accounts for the lack of cellular uptake.

Colocalization experiments with LysoTracker Green (LTG) confirmed that most of the fluorescence observed in intracellular vesicles along the cytoplasm in the case of **ZnPc-NCs** was associated with lysosome accumulation (Figure S23). Pearson and Manders coefficients were calculated by using the 633 nm excitation wavelength to quantify the degree of lysosomal colocalization. As shown in Table S6, the high M1 coefficient (0.677) clearly indicates that a high percentage of the fluorescence signal coming from the **ZnPc-NCs** compound was overlapping the signal from LTG-stained lysosomes. Unfortunately, the poor fluorescence signal produced by **Ir-ZnPc-NCs** hampered the possibility of calculating the colocalization coefficients.

### Photobiological Studies

#### Photocytotoxicity in Normoxia and Hypoxia

Having demonstrated through spectroscopic techniques that both the **ZnPc** and the corresponding Ir(III) conjugate can sensitize Type-I and Type-II ROS upon red light irradiation and that the NCs’ formulations readily internalize into living cells, we focused on investigating their photocytotoxicity toward cancer cells. For this purpose, HeLa cells were incubated with either free or encapsulated compounds in the dark for 1 h. Cells were then either kept in the dark or irradiated for 1 h at 630 nm (89 mW·cm^–2^). After a 48 h drug-free recovery period, cell viability was measured by using a colorimetric assay. This allowed us to calculate dark and light IC_50_ values, i.e., the concentration needed to inhibit cell viability by 50%, and the phototherapeutic index (PI), which is the ratio of dark to light IC_50_ value for each compound.

**Figure 4:**
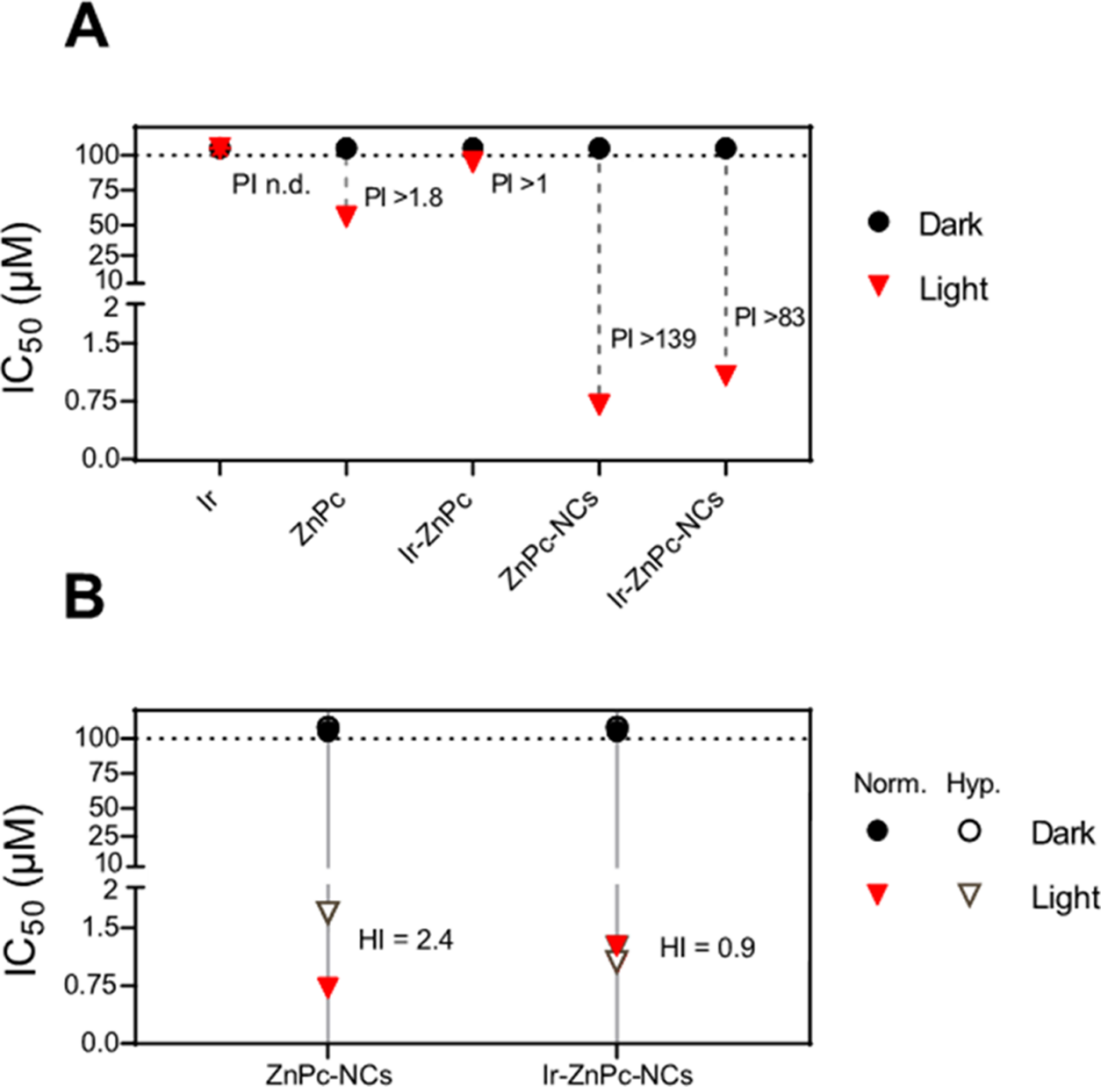
(A) In vitro photocytotoxicity in HeLa cells for **Ir**, **ZnPc**, **Ir-ZnPc**, **Zn-Pc-NCs**, and **Ir–Zn-Pc-NCs** after red light irradiation under normal oxygen conditions. (B) Summary of photocytotoxicity under normoxia (Norm. 21% O_2_, filled symbols) and hypoxia (Hyp. 2% O_2_, unfilled symbols) in HeLa cells for **Zn-Pc-NCs** and **Ir–Zn-Pc-NCs** upon red light irradiation. Light irradiation condition: 630 nm, 1 h, and 89 mW/cm^2^.

None of the tested compounds displayed cytotoxicity under dark conditions of up to 100 μM, which is a desirable trait for PDT agents (Figure 4A and Table 2).

**Table 2:** IC_50_ Values [μM] of Selected Compounds under Dark and after Red Light Irradiation in HeLa Cells^a^

		dark	light	PI^b^	HI^c^
**Ir**	normoxia	>100	>100	n.d.	
**ZnPc**	normoxia	>100	56 ± 4	1.8	
**Ir-ZnPc**	normoxia	>100	95 ± 8	1.0	
**ZnPc-NCs**	normoxia	>100	0.72 ± 0.02	>138.9	
	hypoxia	>100	1.7 ± 0.1	>58.8	2.4
**Ir-ZnPc-NCs**	normoxia	>100	1.2 ± 0.2	>83.3	
	hypoxia	>100	1.1 ± 0.2	>90.9	0.9

a Cells were treated for 2 h (1 h of incubation and 1 h of irradiation at doses of 89 mW cm^–2^ of red light) followed by 48 h of incubation in a drug-free medium under normoxia (21% O_2_) or hypoxia (2% O_2_). Data expressed as mean ± SD from three independent experiments.

b PI = phototoxic index, defined as the ratio of the toxic effect in dark and upon light irradiation; PI = [IC_50_]_dark_/[IC_50_]_630nm_.

c HI = hypoxia index defined as [IC_50_]_hypoxia_/[IC_50_]_normoxia_.

Upon light exposure, nonencapsulated compounds barely induced photocytotoxicity (light IC_50_ > 50 μM), which is in concordance with the negligible intracellular uptake observed by confocal microscopy (Figure 3E,F). In contrast, encapsulated agents exhibited potent photocytotoxicity in the low micromolar range, yielding light IC_50_ values close to 1 μM (Figures 4A, S25, and S26). These results indicated that NCs’ formulation improved the phototoxic effect of both **ZnPc** and **Ir-ZnPc** compounds by a factor of **≈**80. This photopotentiation provided PI values exceeding 139 and 83 for **ZnPC-NCs** and **Ir-ZnPc-NCs**, respectively (Figure 4A).

Since local hypoxia represents a serious impediment for anticancer PDT, the phototoxic action of the nanoencapsulated compounds was then assessed under hypoxic conditions (2% O_2_). As depicted in Figures 4B and S26, both NCs’ formulations were highly photoactive under hypoxia, providing light IC_50_ values that were very similar to those found under normoxia (21% O_2_). This retention of the photoactivity under hypoxia yielded PI values of >59 for **ZnPC-NCs** and >91 for **Ir-ZnPC-NCs** (Table 2). The ability to overcome the photodynamic effect restriction by the lack of oxygen suggests that **ZnPC-NCs** and **Ir-ZnPc-NCs** might operate through Type-I PDT mechanisms, which is coherent with the ROS photogeneration observed via spectroscopic methods (Figure 3C,D). This would explain the capacity of these PS nanoformulations to function under depleted oxygen systems. To illustrate the hypoxia-tolerance of the investigated PDT agents, we calculated a hypoxia index (HI),^32^ defined as the ratio from the light IC_50_ in normoxia to hypoxia (Figure 4B). Analogous to PI, which gives an idea of the differential potency between dark and light and serves as the parameter to optimize an anticancer PDT molecule, the HI provides useful information to optimize the PDT performance of a given PS under varying oxygen levels by comparing potency under hypoxic and normoxic conditions. As such, the HI of **ZnPc-NCs** was 2.4, indicating that the low oxygen tension of hypoxia halved the PDT activity. Remarkably, **Ir-ZnPc-NCs** had a better hypoxia performance (HI = 0.9), which indicated that this nano-PDT agent exerted high photodynamic efficiency regardless of the oxygen tension. Overall, these results suggest that the nanoencapsulated **Ir-ZnPc** conjugate possessed a higher hypoxia-tolerant PDT performance than its **ZnPc** counterpart.

#### Photogeneration of ROS in Cancer Cells

**Figure 5:**
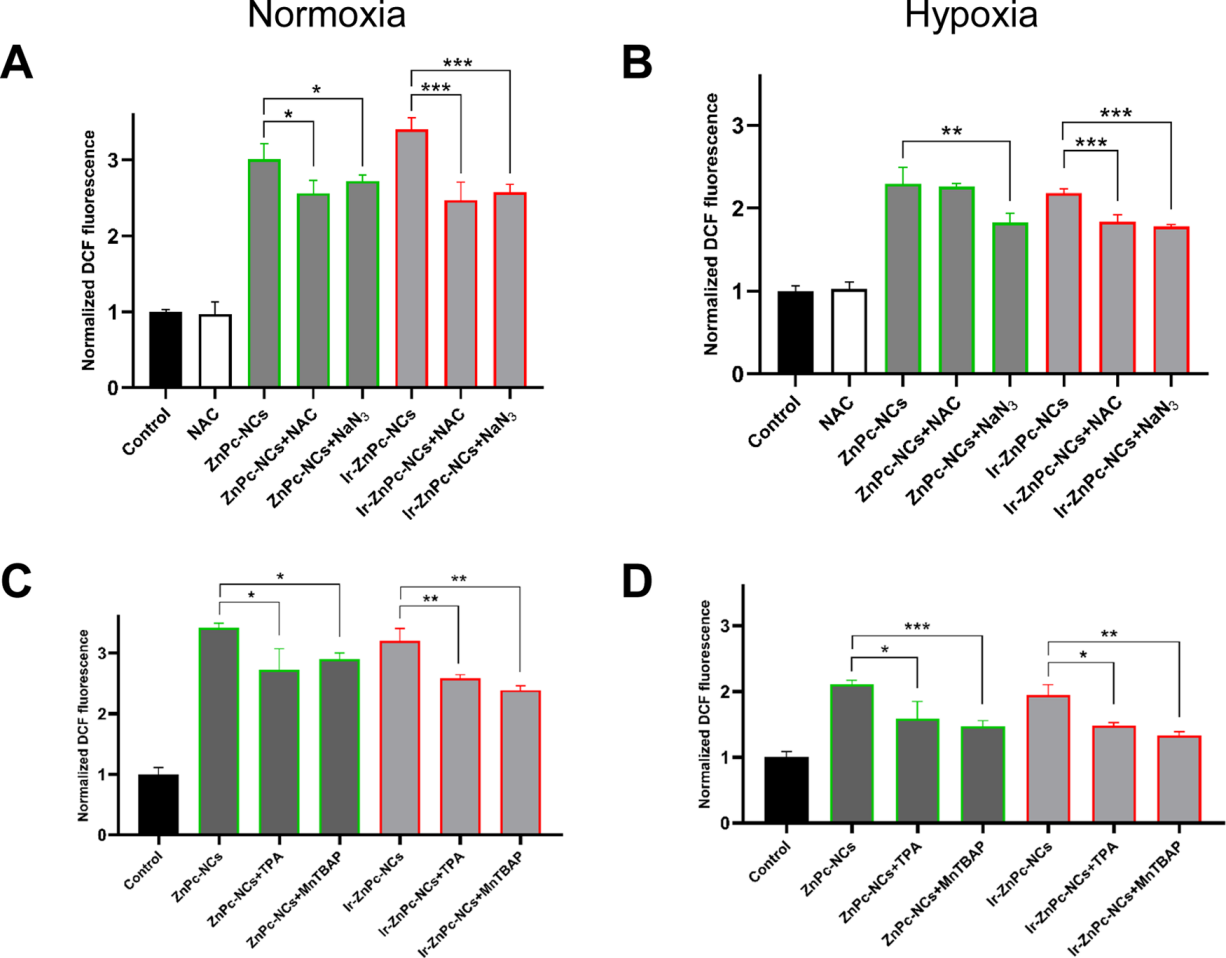
ROS levels in HeLa cells after PDT treatments with **ZnPc-NCs** and **Ir-ZnPc-NCs** (2.5 μM) in the presence of specific scavengers as measured with DCFH-DA probe. (A, B) Cellular ROS levels upon cotreatment with *N*-acetyl cysteine (NAC) and sodium azide (NaN_3_) scavengers in normoxia and hypoxia, respectively. (C, D) Cellular ROS levels upon cotreatment with terephthalic acid (TPA) and MnTBAP in normoxia and hypoxia, respectively. Statistical significance is indicated by *p < 0.05, **p < 0.01, and ***p < 0.001 using unpaired *t* test. Data represented as mean ± SD (*n* = 2 replicates).

Once the photocytotoxicity of the nanoencapsulated compounds against HeLa cells was demonstrated, we investigated the photogeneration of cellular oxidative stress under both normoxia and hypoxia using the ROS probe 2,7-dichlorodihydrofluorescein diacetate (DCFH-DA). As shown in Figures 5 and S27, upon 630 nm light irradiation, a strong fluorescent signal coming from the oxidation of the nonfluorescent DCFH-DA probe into its highly fluorescent form 2′,7′-dichlorofluorescein (DCF) was observed in normoxic cells treated with **ZnPc-NCs** and **Ir-ZnPc-NCs** (approximately 3-fold increase compared to control cells), which indicated an efficient photogeneration of ROS in a cellular environment. To our delight, high levels of ROS were significantly retained under hypoxia, suggesting that the compounds also photogenerated ROS at low oxygen concentrations. With the aim of identifying the specific species generated upon red light irradiation, HeLa cells were cotreated with several selective ROS scavengers following reported protocols.^28,44^ As expected, the general scavenger *N*-acetyl-l-cysteine (NAC) reduced the intracellular ROS levels raised by PDT treatments (Figures 5A,B and S27). In good agreement with the spectroscopic methods, the ^1^O_2_ scavenger sodium azide (NaN_3_) produced a significant decrease in ROS generation (Figures 5A,B and S27). Notably, such a reduction in ROS levels was slightly more pronounced for the **Ir-ZnPc-NCs** than for the **ZnPc-NCs** PDT treatments. No observable alterations in ROS production were observed in cells cotreated with sodium pyruvate, Trolox, and uric acid scavengers, which could rule out the photogeneration of hydrogen peroxide, peroxyl radicals, and peroxynitrite anions, respectively (Figure S28).

The ability of the nanoformulations to produce superoxide (^•^O_2_^–^) and hydroxyl radicals (^•^OH) upon irradiation was also investigated using specific scavengers, namely, the superoxide dismutase mimetic MnTBAP and terephthalic acid (TPA), respectively. As depicted in Figure 5C,D, the addition of such scavengers reduced the formation of global ROS levels under both normoxic and hypoxic conditions, indicating that Type-I and Type-II mechanisms might be simultaneously operating.

#### Photocytotoxicity in 3D Multicellular Tumor Spheroids (MCTS)

**Figure 6:**
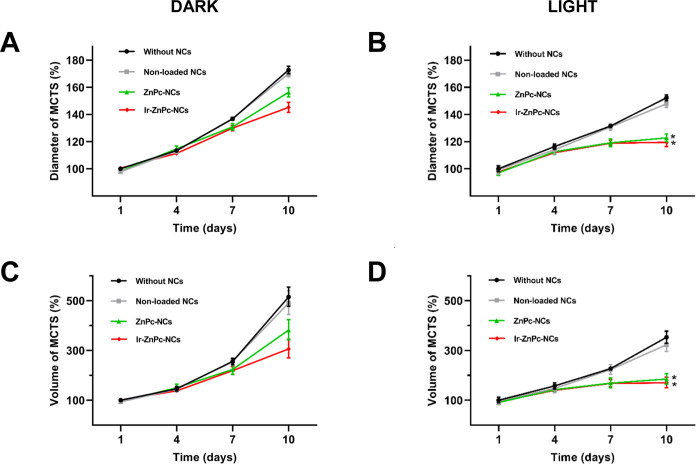
Normalized diameter (A, B) and volume (C, D) of HeLa multicellular tumorspheres (MCTS) over a span of 10 days after treatment with **ZnPc-NCs** or **Ir-ZnPc-NCs** (5 μM) under both dark and light conditions (1 h, 630 nm; 89 mW/cm^2^). The error bars represent the standard deviation (SD) calculated from three replicates, with statistical significance (*p < 0.05) determined by a one-way ANOVA test.

The photoactivity of **ZnPc-NCs** and **Ir-ZnPc-NCs** was investigated on three-dimensional (3D) multicellular tumor spheroids (MCTS) given that these models can reproduce nutrient, drug penetration, and hypoxia gradients, and mimic the growing environment of tumor cells in vivo. HeLa MCTSs were incubated in the dark for 4 h either with nonloaded nanocapsules or with the corresponding **ZnPc** or **Ir-ZnPc** nanoformulations, and were subsequently exposed to red light treatment (1 h, 630 nm; 89 mW/cm^2^) or kept under the dark. After irradiation, the drug-containing medium was removed, and the diameter and volume of the MCTS were monitored over a period of 10 days. Remarkably, upon light irradiation, both **ZnPc-NCs** and **Ir-ZnPc-NCs**-treated MCTS exhibited a significant reduction in both the diameter and volume compared to the nontreated controls (Figures 6 and S29). No effect was observed with nonloaded NCs treatment. The MCTS treated with **ZnPc** or **Ir-ZnPc** nanoformulations continued to display shrinkage in the following days, specifically on day 10, indicating a potent inhibitory effect on tumoral growth. Notably, both nanoformulations demonstrated similar inhibitory effects on 3D MCTS following irradiation, regardless of the drug cargo. Treatments in the dark resulted in comparatively less pronounced alterations in MCTS growth than those observed under light-exposed conditions. These observations indicate that the present **ZnPc-NCs** and **Ir-ZnPc-NCs** behave as potent nano-PDT agents against 3D MCTS models.

**Figure 7:**
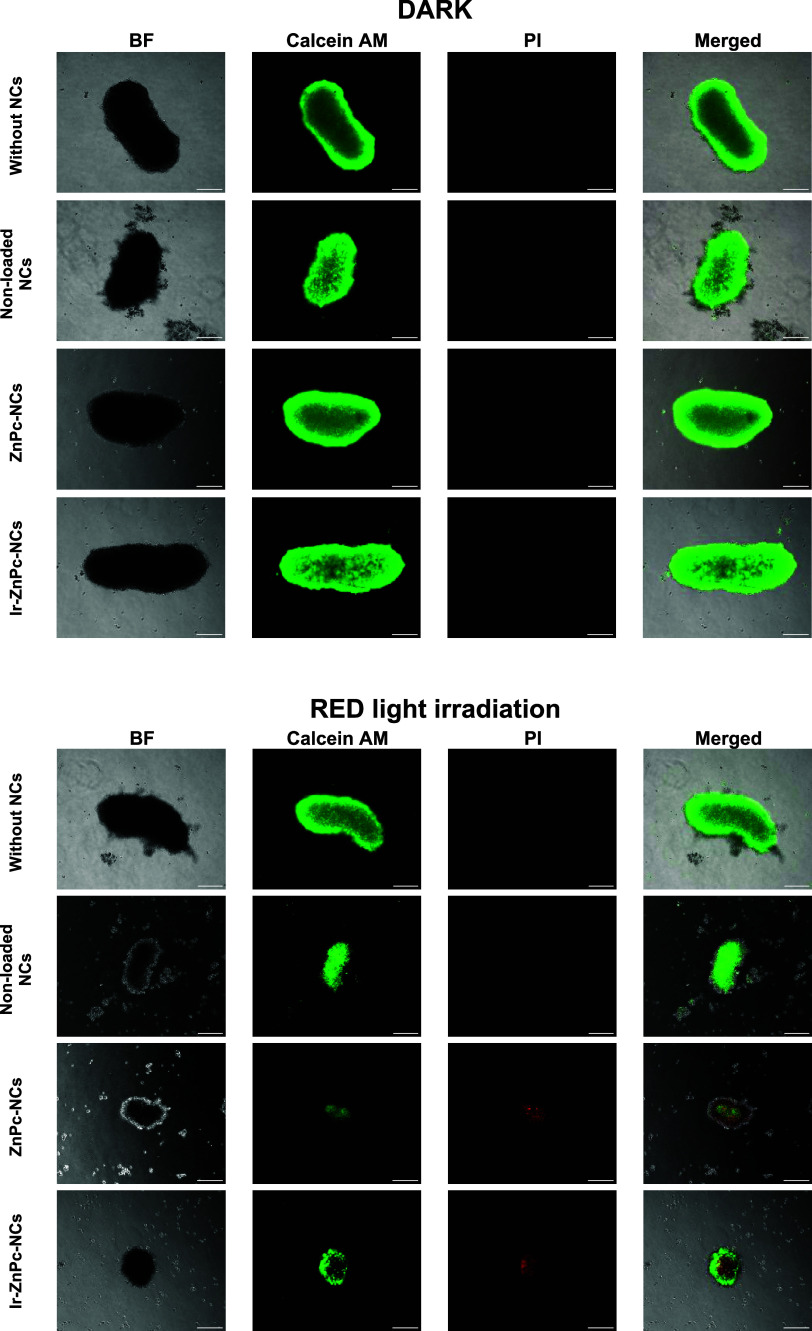
Analysis of HeLa spheroids using confocal microscopy. MCTS were treated with nonloaded nanocapsules, **ZnPc-NCs**, or **Ir-ZnPc-NCs** (50 μM) for 4 h, followed by 1 h red light irradiation and observation after 4 days of incubation, with an additional treatment after the second day. Spheroids were stained with Calcein AM (2 μM) and propidium iodide (2 μg/mL). The same treatments were kept in dark conditions as a control. The scale bar represents 200 μm.

To further investigate the impact of nanoencapsulated compounds on tumor cell viability, treated MCTS were subjected to dual staining with Calcein AM and propidium iodide (Figure 7). The fluorescence microscopy images revealed that both the MCTS treated in the absence of light and those exposed to nonloaded NCs under red light irradiation remained structurally intact. In contrast, MCTS treated with **ZnPc-NCs** and **Ir-ZnPc-NCs** under red light irradiation exhibited a significant reduction in Calcein AM fluorescence activity, coupled with an increase in propidium iodide fluorescence. This indicates a substantial level of cell death within the spheroids, demonstrating the efficacy of the nanoencapsulated compounds in inducing cytotoxicity under red light irradiation.

## Conclusions

In summary, we have successfully conjugated for the first time a zinc phthalocyanine (**ZnPc**) which exhibits excellent absorption into the phototherapeutic window to a highly photostable cyclometalated Ir(III) complex and conveniently explore its application in anticancer PDT. Encapsulating the **Ir-ZnPc** conjugate and the parent **ZnPc** using amphoteric redox-responsive polyurethane-polyurea hybrid nanocapsules was crucial to enable photobiological action since it suppressed some of the main drawbacks of phthalocyanines, including aggregation, low solubility in water, and poor cellular uptake. In addition, both iridium(III) conjugation and nanoencapsulation incremented the photostability of the zinc phthalocyanine. Under normal oxygen conditions, these nanoformulations demonstrated minimal dark toxicity and potent photocytotoxicity within the low micromolar range (PI > 139). Remarkably, both **ZnPc-NCs** and **Ir-ZnPc-NCs** retained their photoactivity under hypoxic conditions; the latter displaying higher hypoxia-tolerant PDT performance (HI = 0.9). The effective photogeneration of intracellular ROS was identified as the source of the high photocytotoxicity of **ZnPc-NCs** and **Ir-ZnPc-NCs** in both normoxia and hypoxia, which points to dual Type-I (superoxide and hydroxyl radicals) and Type-II (singlet oxygen) PDT according to spectroscopic and cell-based assays. Interestingly, the Ir(III) fragment has a clear role in improving the performance of the phthalocyanine scaffold in Type I photosensitizing reactions, which might be attributed to excited-state electron transfer interactions between the redox-active iridium complex and **ZnPc**, as previously found in other conjugates between transition metal complexes and organic fluorophores. Finally, in vitro assays using 3D cellular models confirmed strong antitumor effects from both encapsulated compounds upon 630 nm light exposure. Overall, the prepared ZnPc-based nanoformulations, with their improved photophysical and photobiological properties, hold promise for further evaluation as novel PDT anticancer agents, substantiating their future potential to treat deep-seated hypoxic tumors.

## Experimental Section

### General Materials and Methods

#### NMR and MS

NMR spectra were recorded at room temperature on a BRUKER AVANCE 400 spectrometer (Bruker, Billerica, MA). High-resolution mass spectra were obtained from a Bruker Microflex LRF20 matrix-assisted laser desorption/ionization time-of-flight (MALDI-TOF) using dithranol as the matrix.

#### Infrared Spectroscopy (IR)

IR spectra were registered in a Smart ATR (Nicolet iS10, Thermo Scientific, Raleigh) using a transmittance mode (16 scans) and OMNIC software. For the monitoring of solvent-based samples, one drop was deposited onto the diamond crystal, and the solvent was left to dry by evaporation. IR spectra were recorded from a dry film of the sample for the reaction control after emulsification.

#### pH Measurements

The pH of the emulsion was determined right after the cross-linker was added and at different time intervals until the last polyaddition reaction was complete. All of the determinations were carried out in a pH meter HI 2211 pH/ORP Meter (HANNA Instruments, Eibar, Spain) equipped with a pH electrode Crison 5029 (Crison Instruments, Barcelona, Spain) and a temperature probe.

#### Dynamic Light Scattering (DLS)

The size distribution of the NCs was analyzed on a Zetasizer Nano ZS90 (Malvern, Worcestershire, U.K.) in Milli-Q water at 25 °C at a concentration of 0.5 mg/mL.

#### Transmission Electron Microscopy (TEM)

The morphology of nanocapsules was studied on a TEM Jeol J1010 (Peabody, MA) equipped with a CCD camera (Gatan). A 400-mesh copper grid coated with 0.75% FORMVAR was deposited on 6 μL of a suspension of nanocapsules in water (10 mg mL^–1^) for 25 min. Excess of sample was removed by oblique contact with Whatman filter paper, and the grid was deposited on a drop of uranyl acetate (2% w/w) in water for 30 s. Excess uranyl acetate was removed, and the grid was air-dried for at least 3 h prior to measurement.

#### Zeta-Potential (ζ -Pot)

The ζ-pot of the NCs was analyzed on a Zetasizer Nano ZS90 (Malvern, Worcestershire, U.K.) in Milli-Q water at 25 °C at a concentration of 1 mg/mL, measured at different pH values.

#### Dialysis Purification

The NCs were dialyzed against Milli-Q water for 24 h using a Spectra/Por molecular porous membrane tubing with a 12–14 kDa molecular weight cutoff (MWCO) (Spectrum Laboratories, Rancho Dominguez).

#### Solids Concentration

NCs concentration in the aqueous dispersion was determined in triplicate, leading to dryness using a Digitheat-TFT oven (J.P.Selecta, Barcelona, Spain), with a fixed temperature of 40 °C for 48 h.

#### UV–Visible Spectroscopy

UV–vis measurements were performed in a DINKO UV-6900 spectrophotometer (Dinko Instruments, Barcelona, Spain). Dry THF was chosen as the analysis solvent to solubilize both the polymer and photosensitizers after 48 h drying at 40 °C. UV–visible analyses of nanocapsules were performed after the dialysis process.

#### Determination of Cargo Loading by ICP-MS

To determine the amounts of **Zn**(**Pc**) and **Ir–Zn**(**Pc**) compounds incorporated in the NCs, iridium and zinc were quantified by ICP-MS according to the following procedure. First, a fixed volume of NC emulsion (previously dialyzed) was diluted in 500 μL of concentrated 72% (v/v) nitric acid into Wheaton v-vials (Sigma-Aldrich) and heated in an oven at 373 K for 18 h. The vials were then allowed to cool, and each sample solution was transferred into a volumetric tube and combined with Milli-Q water washings (1.5 mL). Digested samples were diluted 4 times with Milli-Q to obtain a final HNO_3_ concentration of approximately 18% (v/v). Iridium and zinc contents were analyzed on a Nexion350D PerkinElmer instrument at the Centres Cientfics i Tecnolgics of the Universitat de Barcelona. The solvent used for all ICP-MS experiments was 1% HNO_3_-containing Milli-Q water. Iridium and zinc standards were freshly prepared in Milli-Q water with 1% HNO_3_ before each experiment. The concentrations used for the calibration curve were, in all cases, 0, 0.2, 0.4, 1, and 2 ppb. Isotopes detected were ^193^Ir and ^66^Zn. Readings were performed in triplicate for each sample. Rhodium was added as an internal standard at a concentration of 10 ppb in all samples.

Equations 1 and 2 were used to calculate encapsulation efficiency EE (%) and drug loading DL (%):
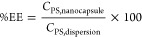
1
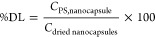
2where *C*_PS,nanocapsule_ is the amount of photosensitizer incorporated into the nanocapsule, *C*_PS,dispersion_ is the total amount of photosensitizer added in the aqueous dispersion, and *C*_dried nanocapsules_ is the total amount of dried nanocapsules. DL and EE values for **ZnPc-NCs** were calculated from the experimental ICP-MS analysis of the zinc content, while the iridium content was used to determine the cargo loading and EE value in the case of **Ir-ZnPc-NCs**.

### Synthetic Procedures

#### Synthesis of Ir(III)-Phthalocyanine Conjugate (**Ir-ZnPc**)

The cyclometalated Ir(III) complexes (**Ir** and **Ir-COOH**)^29^ and zinc phthalocyanines (**ZnPc** and **ZnPc-NH**_**2**_)^40^ were synthesized as previously reported. For the synthesis of Ir(III)-phthalocyanine conjugate, **Ir-COOH** complex (20 mg, 0.02 mmol) and HATU (14 mg, 0.04 mmol) were dissolved in 2 mL of dry DMF and were stirred at 0 °C for 15 min. Then, 200 μL of DIPEA and 47 mg (0.06 mmol) of **ZnPc-NH**_**2**_ were added, and the reaction was stirred at room temperature for 20 h. The solvent was removed under vacuum, and the mixture was purified by silica column chromatography (DCM/MeOH 98:2) yielding 29 mg (91%) of the conjugate. ^1^H NMR (400 MHz, DMSO-*d*_6_): mixture of regioisomers δ(ppm) 13.70 (br, 2H, NH-Ir), 9.47–9.27 (m, 6H), 9.02–8.94 (m, 1H), 8.85 (d, *J* = 8.76 Hz, 1H), 8.67 (d, *J* = 8.71 Hz, 1H), 8.39–8.29 (m, 4H), 8.14–8.08 (m, 2H), 7.99 (d, *J* = 8.58 Hz, 1H), 7.94 (d, *J* = 7.35 Hz, 1H), 7.87–7.84 (m, 2H), 7.73 (m 1H), 7.62 (t, *J* = 7.46, 1H), 7.47–7.40 (m, 2H), 7.28–7.15 (m, 2H), 7.00–6.86 (m, 4H), 6.82–6.77 (m, 2H), 6.65 (br, 2H), 6.31–6.22 (m, 2H), 5.85–5.70 (m, 2H), 5.39 (br, NH amide), 5.08 (br, 2H), 4.69 (br, 2H), 3.90 (br, 2H), 1.77–1.65 (m, 29H), 1.05–0.97 (m, 2H), 0.66 (br, 3H). UV–vis (DMSO) λ_max_/nm (log ε) = 353 (4.98), 612 (4.52), and 679 (5.26). HR-MALDI-TOF (dithranol) *m*/*z* for C_93_H_80_IrN_16_O_2_Zn: calcd 1707.5549; found, 1707.5551.

#### Synthesis of Redox-Responsive Amphiphilic Cationic Prepolymer (**P1**)

2,2′-Dihydroxyethyl disulfide (901.0 mg, 11.68 mequiv), YMER N-120 (12.04 g, 23.18 m equiv), and *N*-(3-(dimethylamino)propyl)-*N*,*N*′-diisopropanolamine (981.3 mg, 8.99 m equiv) were added into a three-neck round-bottom flask equipped with mechanical stirring at room temperature and purged with N_2_. When the mixture was homogeneous, isophorone diisocyanate (8.14 g, 73.24 m equiv) was added into the reaction vessel under gentle mechanical stirring. The polyaddition reaction was kept under these conditions until the NCO stretching band intensity did not change, as monitored by IR spectroscopy. At this point, dry THF (21 mL) was added to the reaction mixture to fluidify the polymer. In parallel, 1,3-diamino-*N*-octadecylpropane (5.99 g, 35.45 m equiv) was dissolved with dry THF (5.23 mL) into another 100 mL three-necked round-bottom flask, which had previously been purged with N_2_. The former reaction mixture was added dropwise onto the latter under half-moon 100 rpm mechanical stirring. The reaction was monitored by IR until the NCO stretching band intensity completely disappeared.

#### Synthesis of ZnPc- and Ir-ZnPc-Loaded Redox-Responsive Amphoteric NCs (**ZnPc-NCs** and **Ir-ZnPc-NCs**)

Isophorone diisocyanate (69.9 mg, 0.63 m equiv) was added into a three-neck round-bottom flask equipped with mechanical stirring, purged with N_2_, and protected from light. In parallel, **ZnPc** or **Ir-ZnPc** (6.8 mg and 6.9 mg, respectively), Neobee 1053 (14.6 mg, 35.73 μmol), polymer **P1** (655.1 mg, 0.07 m equiv) and dry THF (0.25 mL) were mixed in a vial, and subsequently added into the flask and homogenized for 10 min at 150 rpm, while protected from light. At this point, an alkaline aqueous solution of l-lysine was prepared by dissolving 0.93 g l-lysine in 11.37 g of Milli-Q water and adjusting pH to 11.0 by using 3 and 1 M NaOH solutions (total l-lysine concentration 7.56% by wt). The resulting solution (22.84 mg of l-lysine, 0.27 mequiv) was added at 250 rpm to the reaction mixture, and the polyaddition reaction was checked after 15 min by IR spectroscopy. Then, the organic phase was emulsified at 300 rpm with cold Milli-Q water (10.11 g), and finally, a 10% w/w aqueous solution of diethylenetriamine (9.43 mg, 0.27 m equiv) was added in order to generate cross-linked NCs from the nano micelles. The stirring was reduced to 100 rpm. The exact amounts of the reagents are detailed in Table S1. The polyaddition reaction was monitored by IR spectroscopy and pH measurements. Once the NCs were formed, THF was removed from the reactor at 35 °C under vacuum, and the dialysis purification was carried out using a molecular porous membrane tubing with a 12–14 kDa MWCO.

### Photophysical Characterization of the Compounds

For photophysical measurements, all solvents used were of spectroscopic grade. Absorption spectra were registered on a PerkinElmer Lambda 750 S spectrometer with operating software at room temperature. Molar extinction coefficients (ε) were determined by direct application of the Beer–Lambert law, using solutions of the compounds in each solvent with concentrations ranging from 1 to 10 μM. Emission spectra were registered in a Horiba Jobin Yvon Fluorolog 3–22 modular spectrofluorimeter with a 450 W xenon lamp. Measurements were performed in a right-angle configuration using 10 mm quartz fluorescence cells for solutions (10 μM) at room temperature. Emission lifetimes (τ) were measured using an IBH FluoroHub TCSPC controller and a NanoLED pulse diode excitation source (τ < 10 μs); the estimated uncertainty is ±10% or better. Emission quantum yields (Φ) were measured using a Hamamatsu C11347 Absolute PL Quantum Yield Spectrometer; the estimated uncertainty is ±5% or better. CH_2_Cl_2_, DMSO, and water solutions of the samples were previously degassed by bubbling argon for 20 min.

Photostability studies were performed by monitoring the absorbance of a 10 μM DMSO/water (80:20) solution of **Ir**, **ZnPc**, and **Ir-ZnPc** or water solutions of the nanocapsules at room temperature irradiated with a Red Well Plate illuminator photoreactor (Luzchem; Canada) fitter with LED lamps centered at 630 nm (final intensity 89 mW/cm^2^) for 1 h.

### Photochemical Characterization of the Compounds

#### Singlet Oxygen Measurements

Singlet oxygen quantum yields of **ZnPc** and **Ir-ZnPc** were determined in an air-saturated DCM solution (bubbled for 15 min) using 1,3-diphenylisobenzofuran (DPBF) as a chemical trap upon red light irradiation using a high-power LED source (620 ± 15 nm; 130 mW cm^–2^). Upon reaction with singlet oxygen, the fluorescent scavenger DPBF decomposes into a colorless product.^45^ The starting absorbance of DPBF in DCM was adjusted around 1.0 (50 μM), then **ZnPc** or **Ir-ZnPc** were added to the cuvette and their absorbance was adjusted to 0.06 at the light irradiation wavelength (620 nm). Then, the decrease in the absorbance of DPBF at 411 nm was monitored (Figures S18 and S19). The linear relation of the variation in the absorbance (*A*_0_ – *A_t_*) of DPBF at 411 nm against irradiation time was plotted (Figure S20). Singlet oxygen quantum yields were calculated by eq 3
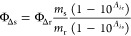
3where Φ_Δr_ is the reference singlet oxygen quantum yield of methylene blue (Φ_Δr_ = 0.57 in DCM),^46^*m* are the slopes, and *A*_λs_ and *A*_λr_ are the absorbances of the compounds and of the reference (methylene blue, MB) at the irradiation wavelength, respectively. The same procedure was used to determine the singlet oxygen quantum yield of **ZnPc-NCs** and **Ir-ZnPc-NCs**. In this case, an air-saturated 1:1 (v/v) mixture of H_2_O and EtOH (bubbled for 15 min) was used as a solvent, and the reference singlet oxygen quantum yield of MB in water was used (Φ_Δr_ = 0.52 in H_2_O).^47,48^

#### Superoxide and Hydroxyl Radical Measurements

Fluorescence emission spectra of the various samples were recorded on a Photon Technology International (PTI) QuantaMaster fluorometer at room temperature. The entrance and exit slits of the excitation and emission monochromators were set at 0.5 mm, giving a spectral bandwidth of 2 nm. The data interval was 1 nm, and the integration time was 0.7 s. All measurements were carried out using a Hellma 1.5 mL PTFE-stoppered fluorescence quartz cuvette (4 clear windows) with a 1 cm path length.

(1) Evaluation of superoxide anion radical generation using DHR123
  DHR123 (10 μM) was added to a solution of the corresponding studied compound (10 μM) in phosphate-buffered saline (PBS) containing 2% DMSO. The resulting solutions were irradiated with a red light LED (620 ± 15 nm, 130 mW·cm^–2^) for the indicated time intervals (0, 1, 2, 3, 4, and 5 min). The fluorescence spectra of the irradiated samples upon excitation at 500 nm were recorded from 510 to 600 nm (DHR123: λ_Ex_ = 507 nm, λ_Em_ = 529 nm). Positive control experiments were carried out using MB as a reference.
(2) Evaluation of hydroxyl radical generation using HPF.

HPF (5 μM) was added to a solution of the corresponding studied compound (10 μM) in PBS containing 2% DMSO. The resulting solutions were irradiated with a red light LED (620 ± 15 nm, 130 mW·cm^–2^) for the indicated time intervals (0, 1, 2, 3, 4, and 5 min). The fluorescence spectra of the irradiated samples upon excitation at 490 nm were recorded from 500 to 600 nm (HPF: λ_Ex_ = 490 nm, λ_Em_ = 515 nm). Positive control experiments were carried out using MB as a reference.

### Confocal Microscopy Studies and Lipophilicity

#### Cell Culture and Treatments

HeLa cells were cultured in DMEM (Dulbecco’s modified Eagle’s medium, Gibco, Life Technologies) supplemented with 10% of fetal bovine or calf serum (Gibco). The cell line was complemented with 100 U·mL^–1^ penicillin–streptomycin mixture (Gibco) and maintained in a humidified atmosphere at 37 °C and 5% of CO_2_.

For cellular uptake experiments and posterior observation under the microscope, cells were seeded on glass dishes (P35G-1.5–14-C, Mattek). 24 h after cell seeding, the cells were incubated at 37 °C for 30 min with free and encapsulated **ZnPc** and **Ir-ZnPc** compounds (10 μM) in supplemented DMEM. Then, the cells were washed three times with DPBS (Dulbecco’s phosphate-buffered saline) to remove the excess of the compounds and kept in DMEM with Hepes (10 mM) and without phenol red for fluorescence imaging.

#### Fluorescence Imaging

All microscopy observations were performed using a Zeiss LSM 880 confocal microscope equipped with a Heating Insert P S (Pecon) and a 63× 1.4 oil immersion objective. The compounds were excited by using the 633 nm laser and detected from 650 to 750 nm. Image analysis was performed using Fiji.^49^ Unless otherwise stated, images are colorized using a Fire lookup table.

Colocalization images using Lysotracker Green DND-26(LTG) were acquired sequentially using a 488 nm laser line, and emission was detected in the 500–550 nm range. Simultaneously, bright-field transmitted light images were acquired. Colocalization analysis was performed using Fiji (ImageJ version 1.53f51). Images were filtered by Median and Gauss filters with a radius of 1 in both cases. Then, the background was subtracted using a Rolling Ball of 10. Finally, colocalization was analyzed using the JaCoP plugin.^50^ Results obtained from the colocalization analyses are summarized in Table S6.

#### Lipophilicity

Distribution coefficients between octanol and water (*K*_O/W_) and log *P* values of compounds **Ir**, **ZnPc**, and **Ir-ZnPc** were calculated using the “shake-flask” method (adapted from refs (32,51)). To this end, solutions of the studied compounds in Milli-Q H_2_O-saturated *n-*octanol (4 mL, final concentration 10 μM for **Ir** or 2.5 μM for **ZnPc** and **Ir-ZnPc**) were prepared in centrifuge tubes from a 10 mM stock solution in DMSO. The solutions were sonicated for 5 min in an ultrasonic bath, and a 2 mL aliquot of each solution was reserved in another centrifuge tube. To the remaining 2 mL of the solutions was added an equal volume of octanol-saturated Milli-Q H_2_O, and the resulting mixtures were vigorously shaken in a vortex for 15 min. Then, the octanol/water mixtures were centrifuged at 7800 rpm for 5 min to separate the phases. The UV–vis absorption spectra of the octanol phases, as well as those of the reserved aliquots were registered using a Jasco V-550 UV–vis spectrophotometer. Log *P* values were calculated according to eq 4
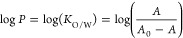
4where *A*_0_ refers to the absorbance of the reserved aliquots of the compounds at their maximum absorption wavelengths (Table S1, λ_Abs_(**Ir**) = 303 nm, λ_Abs_(**ZnPc**) = 678 nm, λ_Abs_(**Ir-ZnPc**) = 678 nm) and *A* is the absorbance of the octanol phase of the corresponding octanol/water mixtures at the same wavelengths.

### Photobiological Studies

#### Phototoxicity Evaluation

HeLa cells were cultured in 96-well plates at a density of 5 × 10^3^ cells/well in complete medium and incubated for 24 h at 37 °C and 5% CO_2_ in a humidified incubator. Hypoxic environment was set up at 2% O_2_. Cell medium was removed by suction and serial dilutions of tested compounds in cell culture media were added at final concentrations in the range of 0 to 100 μM in a final volume of 100 μL/well (%v/v DMSO below 0.4%). Alternatively, water solutions of the encapsulated compounds were further diluted in cell culture media and added directly to cell plates. After 1 h incubation with the compounds, light irradiation was applied using Red Well Plate illuminator photoreactor (Luzchem; Canada) fitter with LED lamps centered at 630 nm (final intensity 89 mW/cm^2^) for 1 h. Dark control analogues were directly kept in the dark for 2 h. Cells were then washed with PBS and fresh media was added for a drug-free cell recovery period of 48 h. Cell media was then removed and wells were loaded with 50 μL of MTT solution (1 mg/mL) for an additional 4 h, then removed, and 50 μL of DMSO was added to solubilize the purple formazan crystals formed in active cells. The absorbance was measured at 570 nm using a microplate reader (FLUOstar Omega), and the IC_50_ values were calculated based on the inhibitory rate curves using eq 5
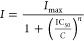
5where *I* represents the percentage inhibition of viability observed, *I*_max_ is the maximal inhibitory effect, IC_50_ is the concentration that inhibits 50% of maximal growth, *C* is the concentration of the treatment, and *n* is the slope of the semilogarithmic dose–response sigmoidal curves. The nonlinear fitting was performed using SigmaPlot 14.0 software. Two independent experiments were performed with triplicate points per concentration level (*n* = 3).

#### Photogeneration of ROS

HeLa cells were seeded on 12-well plates at a density of 2 × 10^5^ cells/well and incubated for 24 h under normoxia (21% O_2_) or hypoxia (2% O_2_) in a humidified CO_2_ incubator. Then cells were cotreated with 2.5 μM of the compounds and a variety of ROS scavengers for 1 h. *N*-Acetyl cysteine (NAC, 5 mM) was used as a general radical scavenger. Hydrogen peroxide (H_2_O_2_) was scavenged using sodium pyruvate (NaPyr, 10 mM), whereas sodium azide (NaN_3_, 5 mM) was used for singlet oxygen (^1^O_2_). Peroxyl radical (ROO^•^) and peroxynitrite anion (ONOO^–^) production was scavenged with Trolox (100 μM) and uric acid (100 μM), respectively. In all cases, treatment was followed with 1 h of incubation in the dark and then 1 h of irradiation with red light. Similarly, MnTBAP (10 μM) and terephthalic acid (20 μM) were used to reduce the formation of superoxide anion (O_2_^•–^) and hydroxyl radicals (^•^OH), respectively. In this case, the cells were irradiated with red light for 30 min. After irradiation, the medium was removed, and cells were stained with 2′,7′-dichlorofluorescein diacetate (DCFH-DA, 10 μM) for 30 min in the dark. Flow cytometry (Fortessa X20) was performed to detect emission at 530 nm after excitation with a blue laser (488 nm). The assay was carried out in at least two independent experiments (*n* = 2 per replicate).

#### Assessment of Photocytotoxicity in 3D Multicellular Tumor Spheroids

To generate HeLa multicellular tumor spheroids (MCTS), 96-well Corning microplates with an ultralow attachment surface coating were used. Initially, a single suspension of HeLa cells, consisting of 6 × 10^3^ cells per well, was prepared in complete DMEM and then carefully distributed into the designated wells. These plates were subsequently covered and transferred to an incubator maintained at 37 °C with a 5% CO_2_ atmosphere. Within a period of 3–4 days, the suspended cells self-assemble into uniform and compact MCTS, each with an average diameter of 400 μm under the specified culture conditions. On day 1, the MCTS were exposed to **ZnPc-NCs** or **Ir-ZnPc-NCs** at a concentration of 50 μM for 4 h, followed by red light irradiation for 1 h. Similarly, nonloaded NCs or cell medium was used as a control. Another set of spheroids underwent the same treatments but was kept in the dark as a control. Subsequently, treatments were replaced with fresh cell media, and every 3 days, the treatments were repeated. Over a span of 10 days, the development and characteristics of the MCTS were meticulously observed and analyzed using a DMi1 inverted phase contrast microscope (Leica Microsystems).

#### Live/Dead Viability/Cytotoxicity in 3D MCTS

MCTS were treated with nonloaded nanocapsules, **ZnPc-NCs** and **Ir-ZnPc-NCs** at a concentration of 50 μM for 4 h, followed by red light irradiation for 1 h. Following irradiation, the treatments were replaced with fresh cell culture media, and the spheroids were incubated in the dark. After 2 days, the same treatments were repeated, followed by an additional 2 days of incubation. Thereafter, the spheroids were washed and stained with Calcein AM (2 μM) and propidium iodide (2 μg/mL) for 30 min at 37 °C in a 5% CO_2_ atmosphere. Fluorescence images of the MCTS were then captured using a Zeiss Axio Observer 7 inverted fluorescence microscope.

## Supporting Information Available

General methods and analytical techniques; synthesis and characterization of **ZnPc** and **Ir-ZnPc** (NMR, MS) and their nanoencapsulated forms (IR, DLS, TEM, ζ-potential); and their photophysical, photochemical, and biological (confocal microscopy, photocytotoxicity, and ROS photogeneration) characterization (PDF)
