# Supplementary material for: A Nanoencapsulated Ir(III)-Phthalocyanine Conjugate as a Promising Photodynamic Therapy Anticancer Agent
Source: ACS Appl Mater Interfaces. 2024 Jul 23;16(30):38916–30. doi: 10.1021/acsami.4c05181 (PMC11299137; doi:10.1021/acsami.4c05181)
Supplement: Supplementary file 1 — am4c05181_si_001.pdf [file am4c05181_si_001.pdf]

Supporting Information

A nanoencapsulated Ir(III)-phthalocyanine conjugate as a promising photodynamic therapy anticancer agent

Joaquín Bonelli,[1,2,#] Enrique Ortega-Forte,[3,#] Gloria Vigueras,[3,#] Jorge Follana-Berná,[4,#] Pezhman Ashoo,[3,#] Diego Abad-Montero,[1] Neus Isidro,[2] Marta López-Corrales,[1] Adrián Hernández,[4] Javier Ortiz,[4] Eduardo Izquierdo-García,[1] Manel Bosch,[5] Josep Rocas,[2] Ángela Sastre-Santos,[4,*] José Ruiz,[3,*] Vicente Marchán[1,*]

[1] Departament de Química Inorgànica i Orgànica, Secció de Química Orgànica, Universitat de Barcelona (UB), and Institut de Biomedicina de la Universitat de Barcelona (IBUB), Martí i Franquès 1-11, E-08028 Barcelona, Spain. Email: vmarchan@ub.edu

[2] Ecopol Tech SL. Nanobiotechnological Polymers Division, R&D Department, El Foix Business Park, Indústria 7, E-43720 L'Arboç del Penedès, Tarragona, Spain

[3] Departamento de Química Inorgánica, Universidad de Murcia, and Institute for Bio-Health Research of Murcia (IMIB-Arrixaca), E-30071 Murcia, Spain. Email: jruiz@um.es

[4] Área de Química Orgánica, Instituto de Bioingeniería, Universidad Miguel Hernández, Avda. de la Universidad s/n, E-03203 Elche, Spain. E-mail: asastre@umh.es

[5] Unitat de Microscòpia Òptica Avançada, Centres Científics i Tecnològics, Universitat de Barcelona, Av. Diagonal 643, E- 08028 Barcelona (Spain)

# These authors contributed equally

Table of contents

1. Synthetic procedures

    1.1. Synthesis of iridium(III)-phthalocyanine conjugate (**Ir-ZnPc**) S3

    1.2. Synthesis of **ZnPc**- and **Ir-ZnPc**-loaded redox responsive amphoteric NCs (**ZnPc-NCs** and **Ir-ZnPc-NCs**) S5

2. Characterization of prepolymer **P1**, **ZnPc-NCs** and **Ir-ZnPc-NCs**.

    2.1. Infrared Spectroscopy S6

    2.2. NCs concentration, **ZnPc** and **Ir-ZnPc** loading and encapsulation efficiency S8

    2.3. Average size of NCs S9

    2.4. Transmission electron microscopy (TEM) S10

    2.5. Z-potential of **ZnPc-NCs** and **Ir-ZnPc-NCs** S11

3. Photophysical characterization of the compounds S12

4. Singlet oxygen measurements S16

5. Superoxide and hydroxyl radical measurements S19

6. Confocal microscopy studies and lipophilicity S21

7. Photobiological studies

    7.1. Phototoxicity evaluation S23

    7.2. Photogeneration of ROS S25

    7.3. Assessment of photocytotoxicity in 3D multicellular spheroids S27

1. Synthetic procedures

1.1. Synthesis of Ir(III)-phthalocyanine conjugate (Ir-ZnPc)

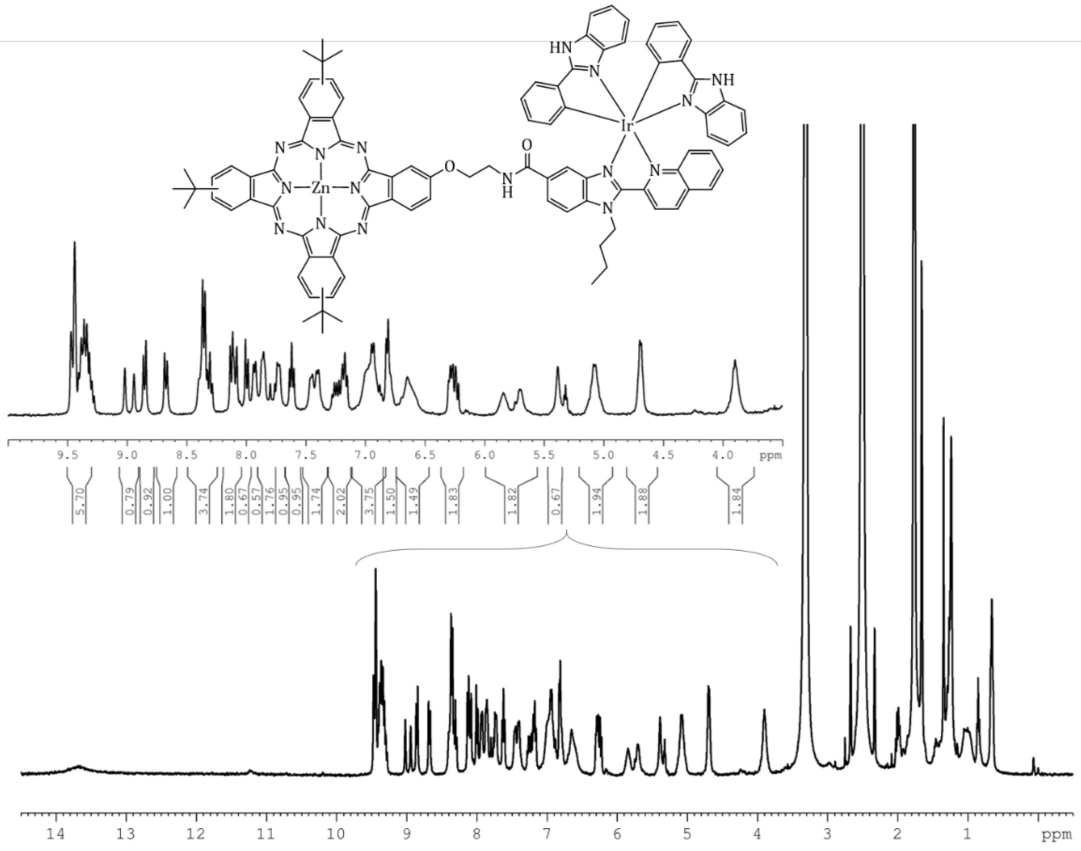

Figure S1. ^{1}H NMR spectrum of **Ir-ZnPc** in DMSO-d_6.

A)

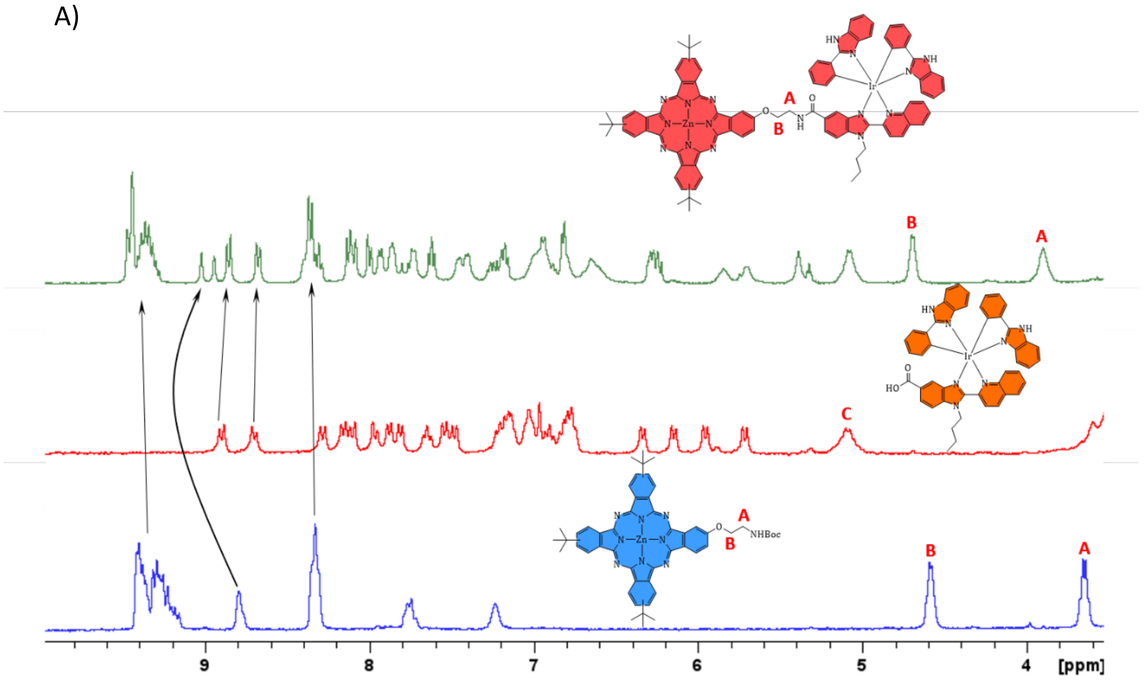

B)

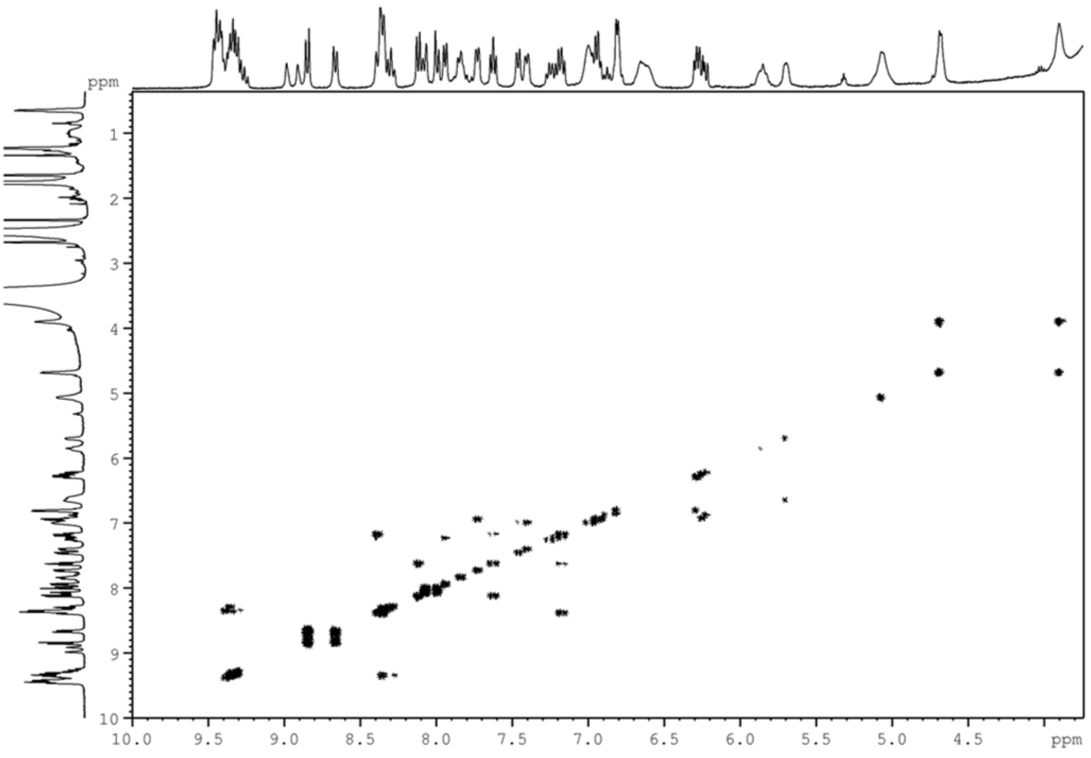

Figure S2. A) Comparison of the ^{1}H NMR spectra of **Ir-ZnPc** with those of the reference compounds (**Ir-COOH** and **ZnPc**) in DMSO-d_6. B) COSY spectrum of **Ir-ZnPc** in DMSO-d_6.

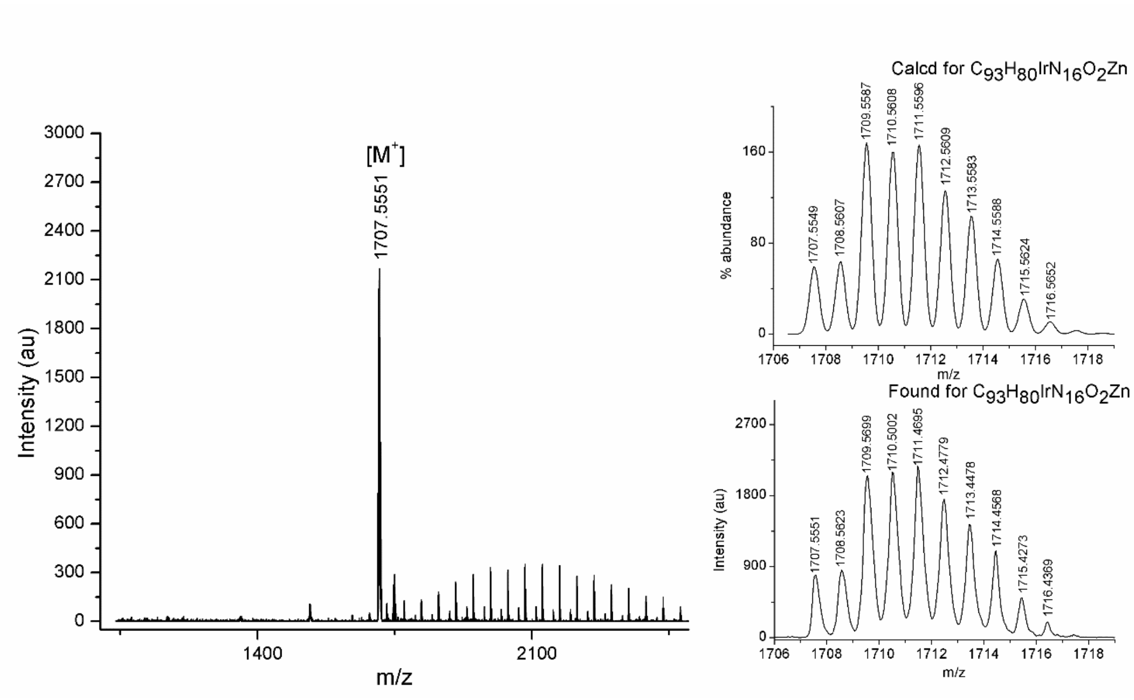

Figure S3. HR-MALDI-TOF MS of **Ir-ZnPc** conjugate.

1.2. Synthesis of ZnPc- and Ir-ZnPc-loaded redox responsive amphoteric NCs (ZnPc-NCs and Ir-ZnPc-NCs)

Table S1. Amount of reagents used to prepare **ZnPc-NCs** and **Ir-ZnPc-NCs**.

**Compound**	**Amount**	**Equivalents or mols**
**IPDI**	69.7 mg	0.63 meq
**ZnPc or Ir-ZnPc**	6.8 mg ZnPc or 6.9 mg Ir-ZnPc	7.73 µmol ZnPc or 4.09 µmol Ir-ZnPc
**Neobee 1053 (GTCC)**	26.3 mg	56.61 µmol
**Polymer (P1)**	812.5 mg	0.09 meq
**Dry THF**	1 mL	–
**L-lysine**	20.9 mg	0.25 meq
**Milli-Q water**	5.93 g	–
**DETA**	7.8 mg	0.23 meq

2. Characterization of prepolymer P1, ZnPc-NCs and Ir-ZnPc-NCs.

2.1. Infrared Spectroscopy

The polymerization reaction for the synthesis of prepolymer P1 as well as for the nanoencapsulation process were monitored by IR spectroscopy by taking advantage of the very clear and characteristic stretching band of the NCO group at 2280-2230 cm^{-1}. On the one hand, consecutive IR spectra for P1 synthesis are represented in Figure S1, in which the green line corresponds to the first sample recorded at the beginning of the reaction, the red line corresponds to the end of the first step, and blue line represents the spectrum once the reaction between NCO groups and the diamine was completed. As shown in Figure S1, the NCO asymmetric stretching band at 2252 cm^{-1} was very sharp and intense at the beginning of the reaction (green line). However, the intensity of this band decreased significantly at the end of the first step which involved the reaction between the diols and the diisocyanate (red line). Meanwhile, the intensities of the CO stretching band at 1719 cm^{-1}, the CN stretching band at 1537 cm^{-1}, the NCOO/COC asymmetric stretching band at 1240 cm^{-1} increased. Overall, the IR spectra registered during the first step of the synthesis confirmed polyurethane bond formation along with NCO consumption. Once the diamine was added, during the second step of the polymer synthesis (blue line), the NCO stretching band at 2252 cm^{-1} disappeared instantaneously, which was explained by the high reactivity of the amines. Simultaneously, other characteristic bands appeared or changed, such as a new stretching band at 1634 cm^{-1}, which was associated to the carbonyl of urea bonds and a new wagging band at 908 cm^{-1} corresponding to the free secondary amine, which also confirmed polyurea formation.

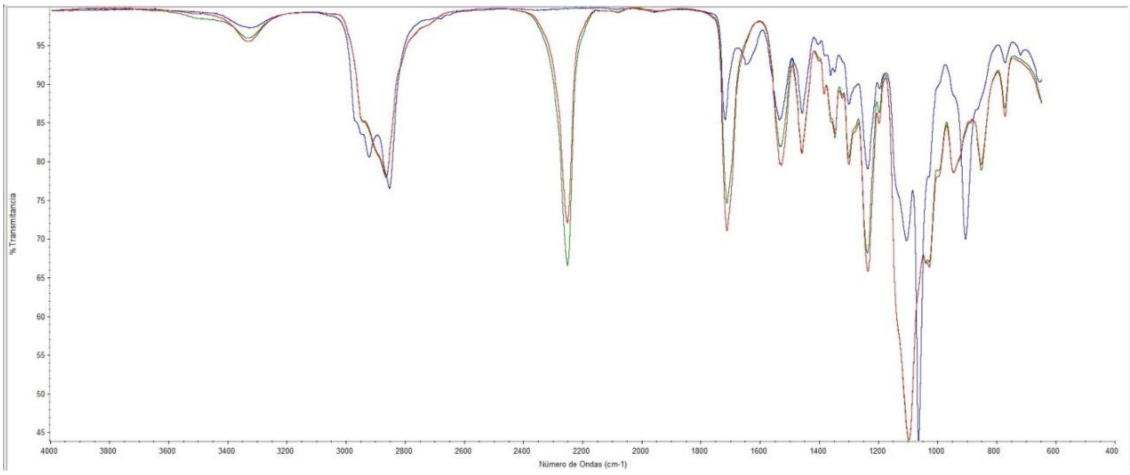

Figure S4. Consecutive IR spectra recorded during the synthesis of P1.

On the other hand, Figure S2 shows the consecutive IR spectra during the synthesis of **Ir-ZnPc-NCs**, where the cyan line represents the sample 30 min after mixing the P1 prepolymer with the cargo and the diisocyanate. This initial step was the reactivation of the polymer and its conversion to an NCO-reactive entity. The green line corresponds to the step in which L-lysine sodium salt was added and reacted with the activated polymer. A decrease on the intensity of the NCO stretching band at 2255 cm^{-1}, concomitantly with an increase of the carbonyl and C-N stretching bands, confirmed urea bond formation (1642 cm^{-1} and 1532 cm^{-1}, respectively). Finally, the red line corresponds to the complete crosslinking of NCO-groups after addition of the triamine, which caused the instantaneous disappearance of the NCO stretching band and an increase of the intensity of the urea-associated bands because of the rapid reaction between remaining NCO groups and the polyamine.

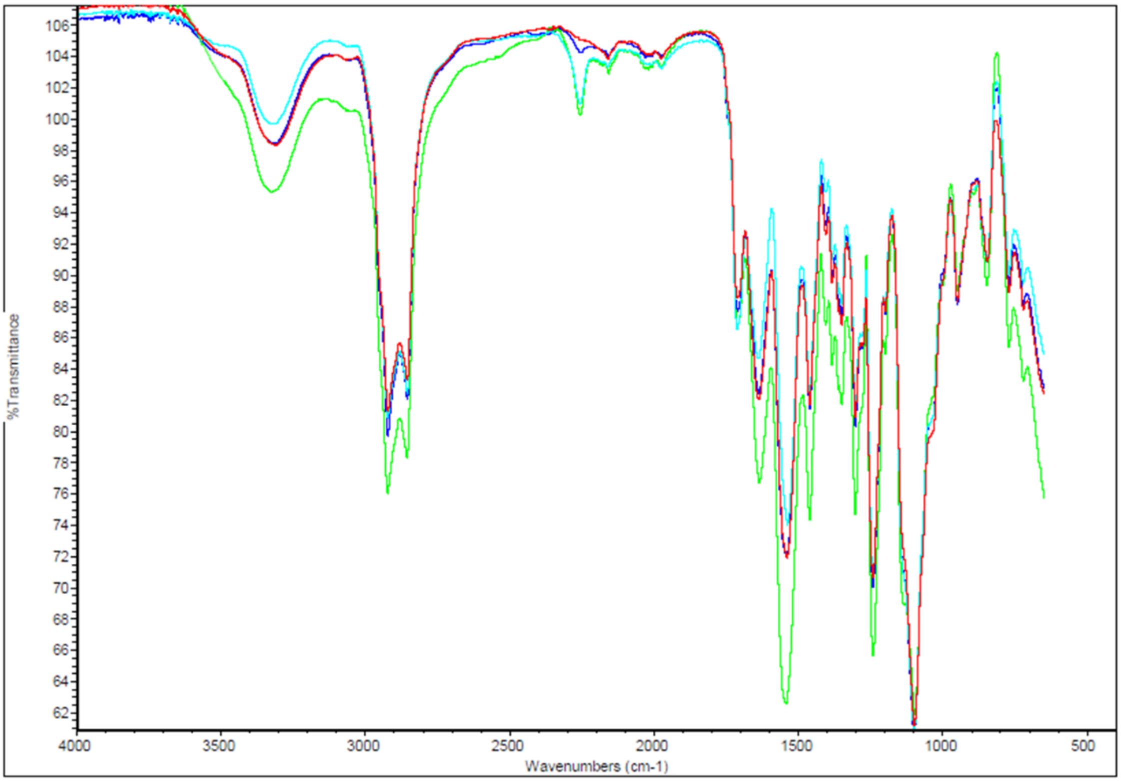

Figure S5. Consecutive IR spectra recorded during the encapsulation process of **Ir-ZnPc**.

2.2. NCs concentration, ZnPc and Ir-ZnPc loading and encapsulation efficiency

The concentration of NCs in the final emulsions (mg/mL) was quantified with a solids concentrator. Cargo loading (DL) and Encapsulation Efficiency (EE) were determined by ICP-MS analysis. The DL and EE parameters for **NC-GTCC**, **ZnPc-NCs** and **Ir-ZnPc-NCs** are indicated in Table S2.

Table S2. DL and EE parameters for **ZnPc-** and **Ir-ZnPc**-loaded NCs.

	**[NCs] (mg/mL)**	**PS Loading (DL, µM)**	**Encapsulation Efficiency (EE, %)**
**NC-GTCC**	42.9 ± 1.6	-	-
**ZnPc-NCs**	30.2 ± 0.1	621.7 ± 93.0 µM	64.3 %
**Ir-ZnPc-NCs**	62.4 ± 1.1	256.5 ± 20.5 µM	50.2%

2.3. Average size of NCs

The particle size distribution of the NCs was measured by dynamic light scattering (DLS). The experimental values and the number average are displayed in Table S3 and the size distribution by number of the NCs is graphically displayed in Figures S6 and S7.

Table S3. Hydrodynamic diameter average of **ZnPc**- and **Ir-ZnPc**-loaded NCs.

Compound	Experimental values (nm)	Average ± SD (nm)
**ZnPc-NCs**	12.29	11.4 ± 1.1
	11.74	
	10.19	
**Ir-ZnPc NCs**	13.44	15.2 ± 1.6
	16.50	
	15.55	

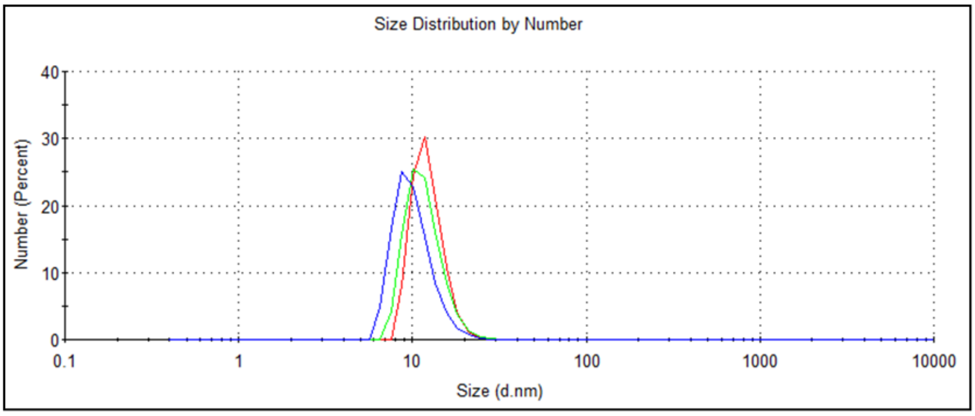

Figure S6. Average size of **ZnPc-NCs**.

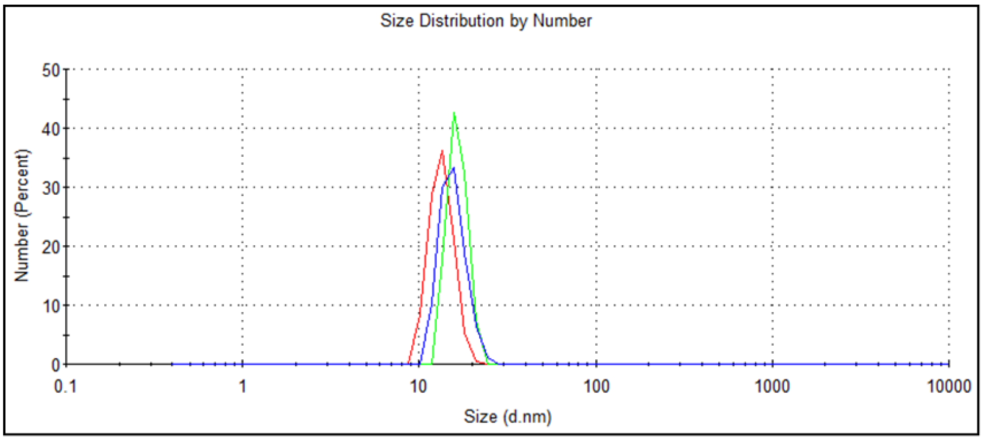

Figure S7. Average size of **Ir-ZnPc-NCs**.

2.4. Transmission electron microscopy (TEM)

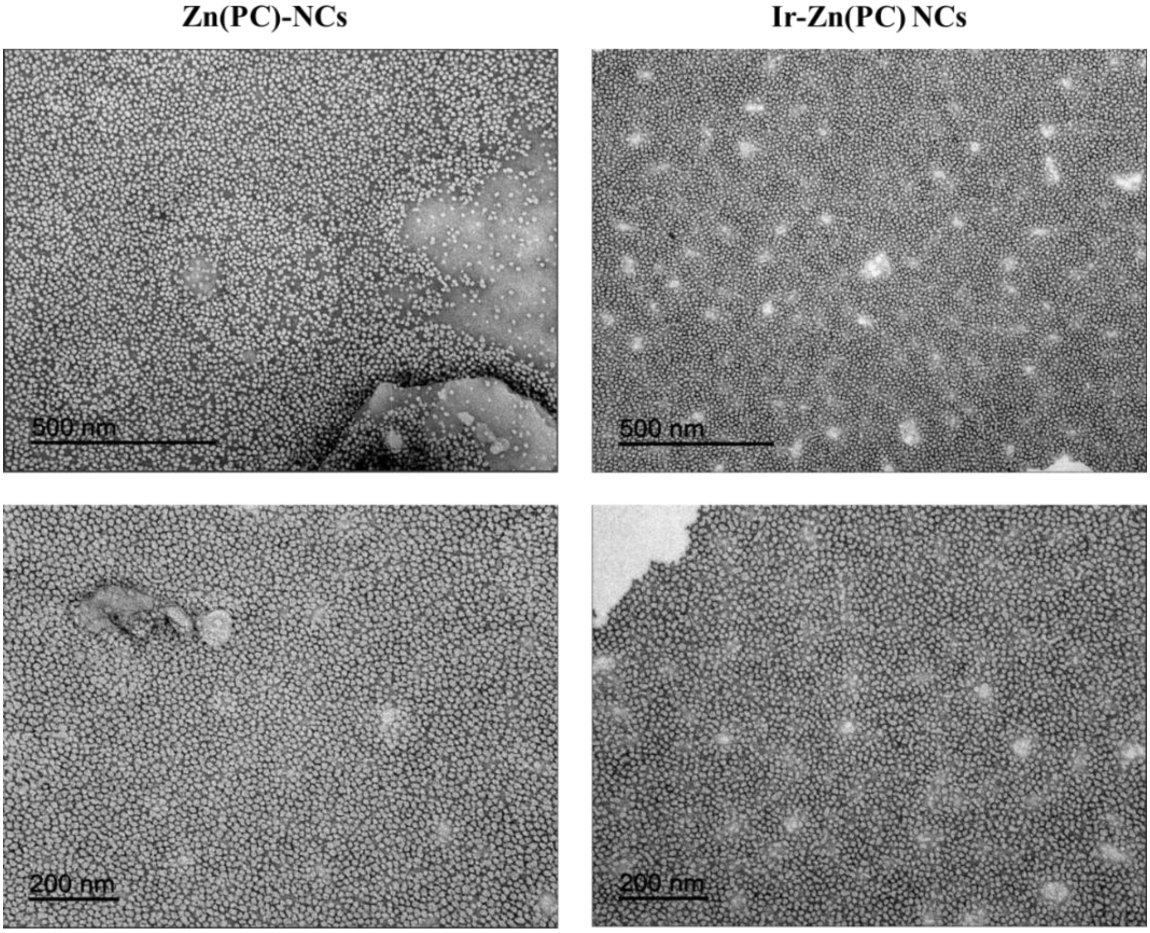

Figure S8. TEM micrographs of **ZnPc-NCs** and **Ir-ZnPc-NCs**.

2.5. Z-potential of ZnPc-NCs and Ir-ZnPc-NCs

As shown in Figures S9 and S10, the Z-potential values (surface charge) of the NCs were determined at different pH values as described in Materials and methods section. Results of Z-potential analyses have been summarized in Table S4.

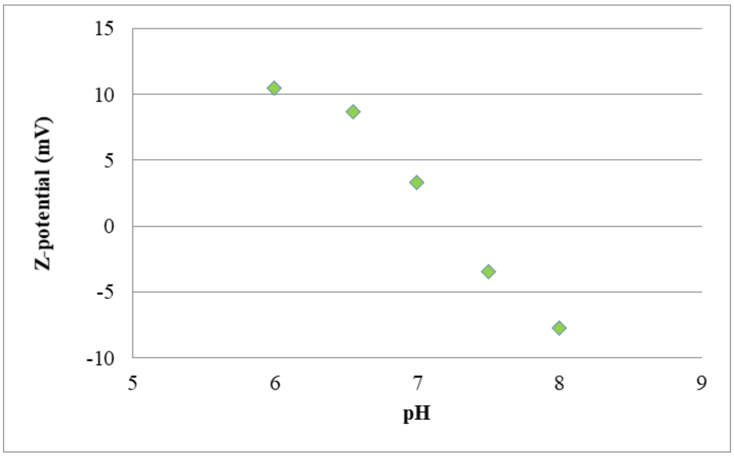

Figure S9. Z-potential values of **ZnPc-NCs** *vs* pH.

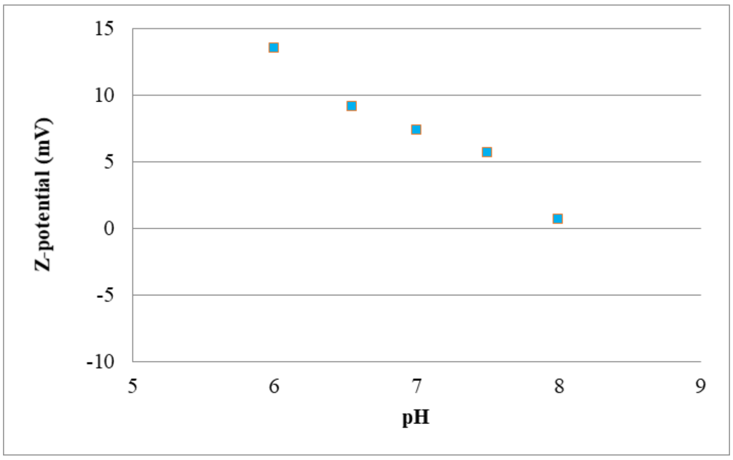

Figure S10. Z-potential values of **Ir-ZnPc-NCs** *vs* pH.

Table S4. Z-potential values (mV) of the samples against pH variation.

**Sample**	**Z-Pot ± SD pH=6.0**	**Z-Pot ± SD pH=6.5**	**Z-Pot ± SD pH=7.0**	**Z-Pot ± SD pH=7.5**	**Z-Pot ± SD pH=8.0**
**ZnPc-NCs**	10.5 ± 0.5	8.7 ± 1.0	3.4 ± 0.6	-3.4 ± 0.4	-7.7 ± 0.3
**Ir-ZnPc-NCs**	13.5 ± 3.6	9.1 ± 1.5	7.2 ± 0.7	5.7 ± 0.5	0.7 ± 0.3

3. Photophysical characterization of the compounds

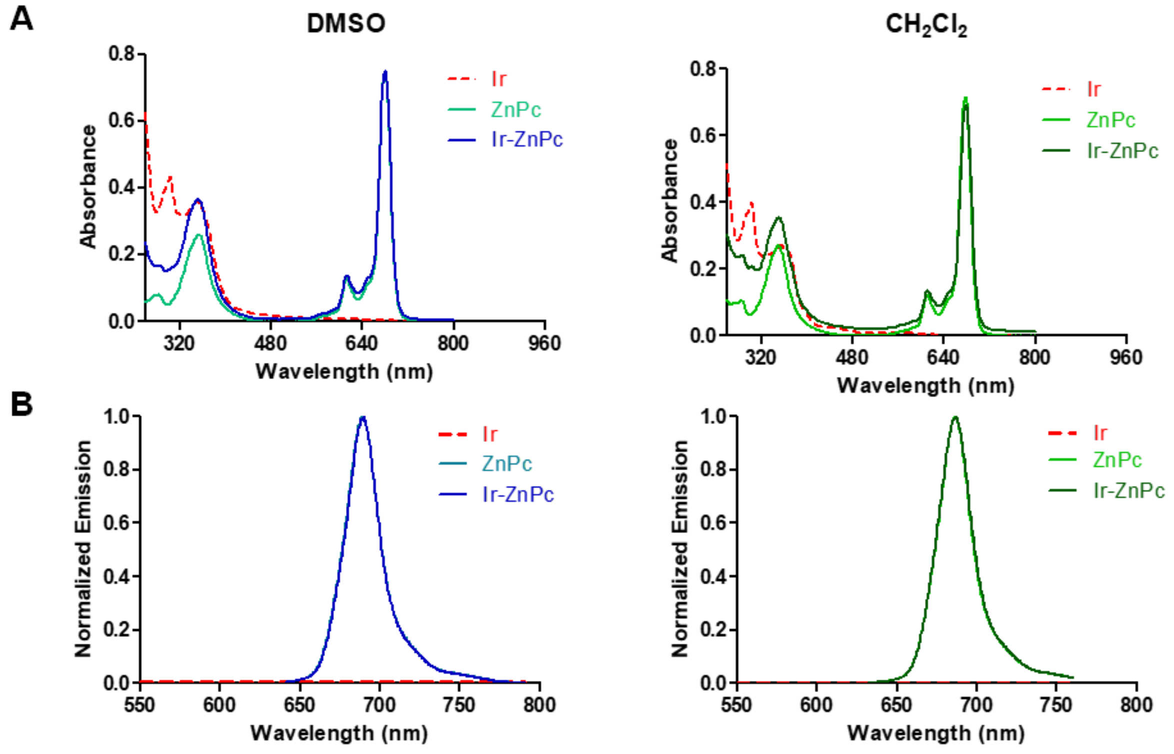

Figure S11. A) UV-visible and B) normalized emission spectra (λ_{exc} = 620 nm) of iridium complex (**Ir**), Zn phthalocyanine (**ZnPc**) and the conjugate (**Ir-ZnPc**) in aerated solutions of DMSO and CH_2Cl_2.

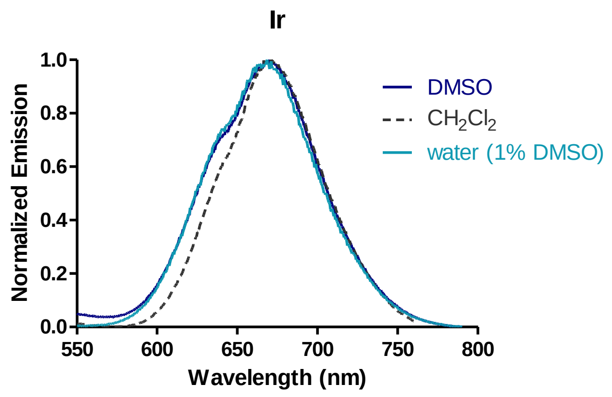

Figure S12. Normalized emission spectra of the Ir(III) complex **(Ir)** in aerated solutions. λ_{exc} = 405 nm.

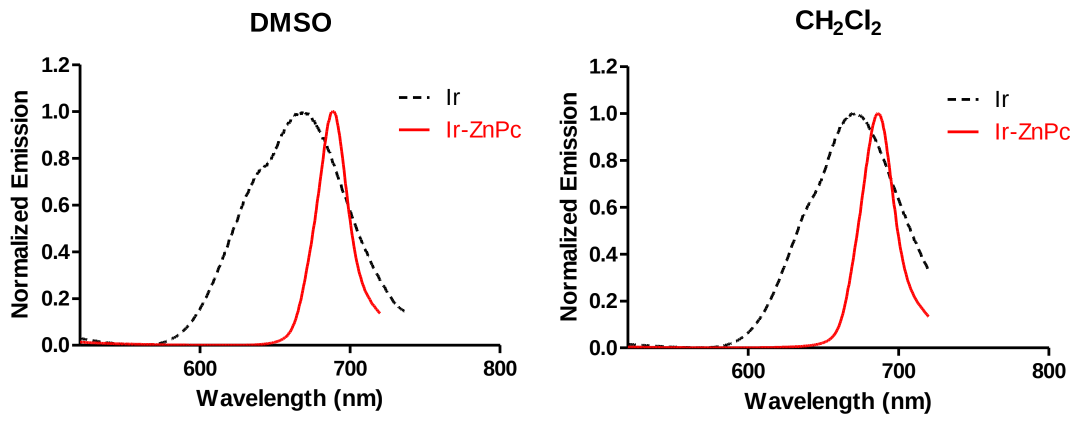

Figure S13. Normalized emission spectra of iridium compound (**Ir**) and conjugate (**Ir-ZnPc**) at λ_{exc} = 370 nm.

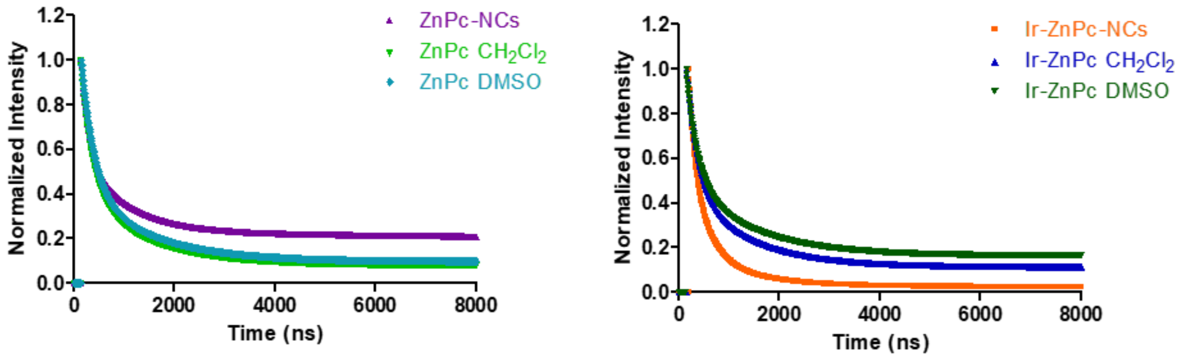

Figure S14. Normalized luminescence decay curves of **ZnPc** and **Ir-ZnPc**.

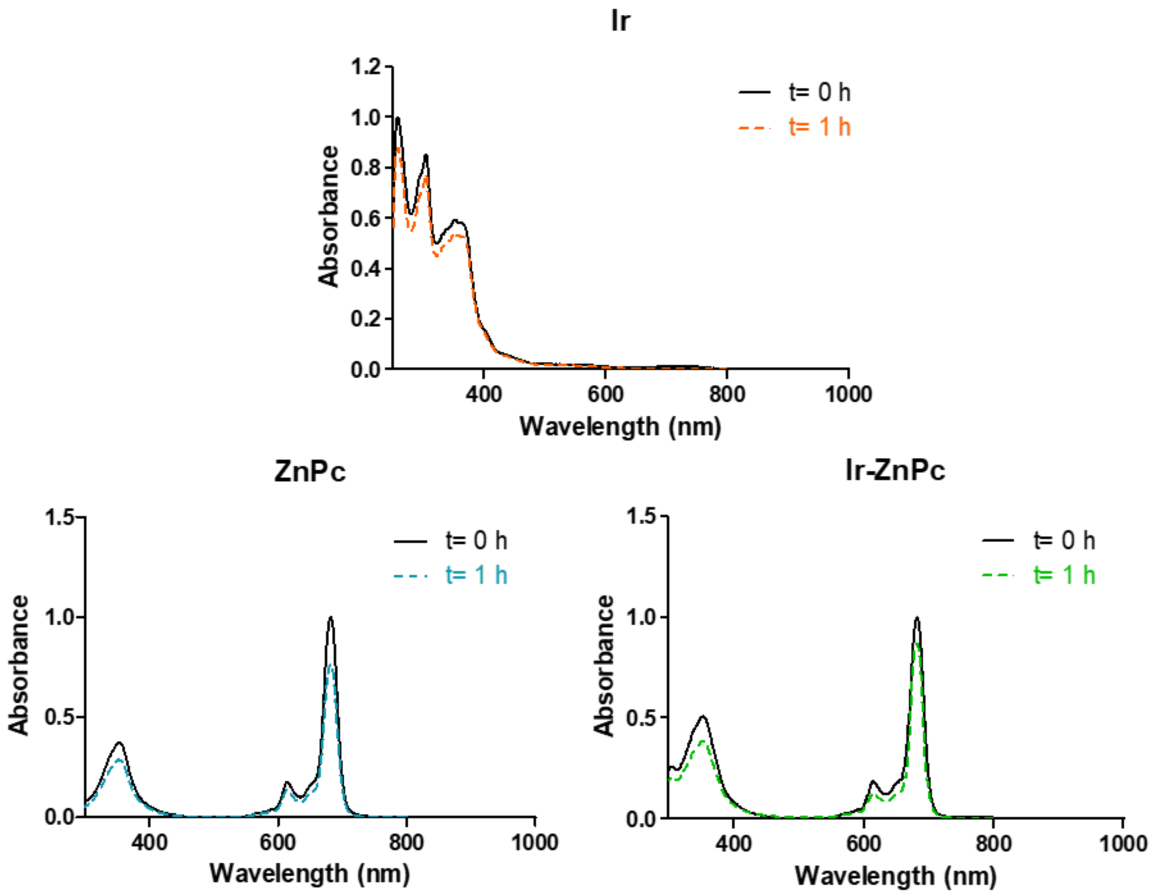

Figure S15. Photostability at 630 nm (89 mW/cm^2) in DMSO/H_2O (80:20) of **Ir**, **ZnPc** and **Ir-ZnPc**.

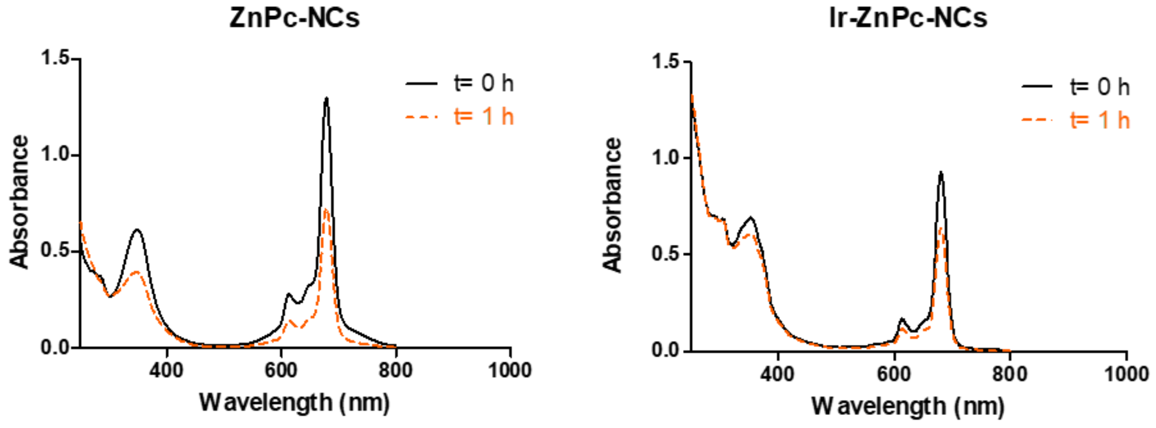

Figure S16. Photostability at 630 nm (89 mW/cm^2) of **ZnPc-NCs** and **Ir-ZnPc-NCs** in water.

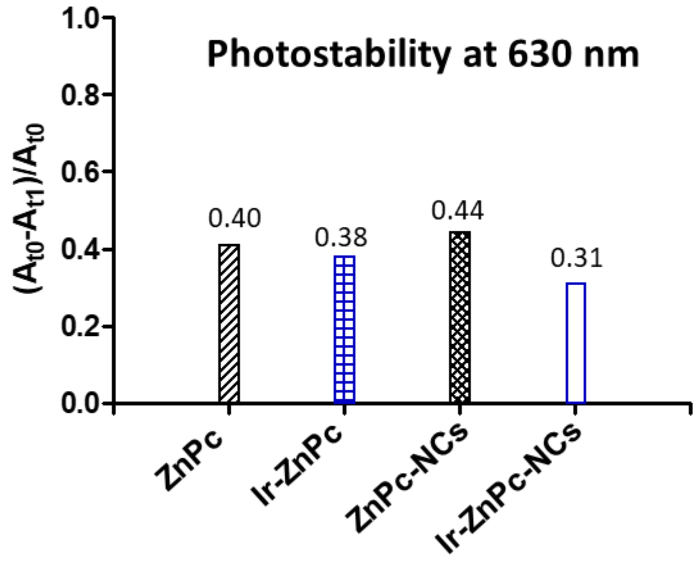

Figure S17. Values of the decrease in the absorbance of the compounds at 678 nm after irradiation at 630 nm (89 mW/cm^2) for 1 h. Data was taken from Figures S15 and S16.

4.- Singlet oxygen measurements

Table S5. Singlet oxygen quantum yield of the investigated compounds.

Compound	Φ_Δ
**ZnPc**	0.61
**Ir-ZnPc**	0.65
**ZnPc-NCs**	0.54
**Ir-ZnPc-NCs**	0.58

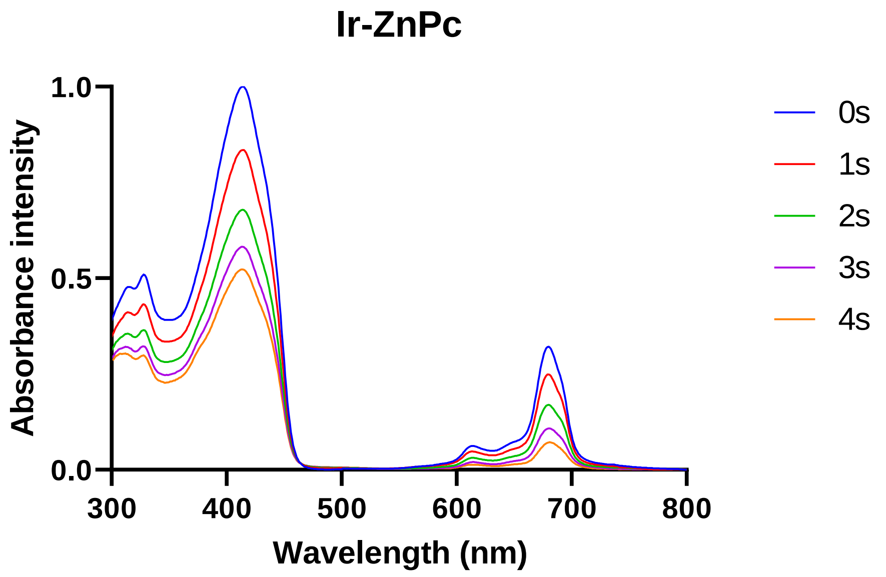

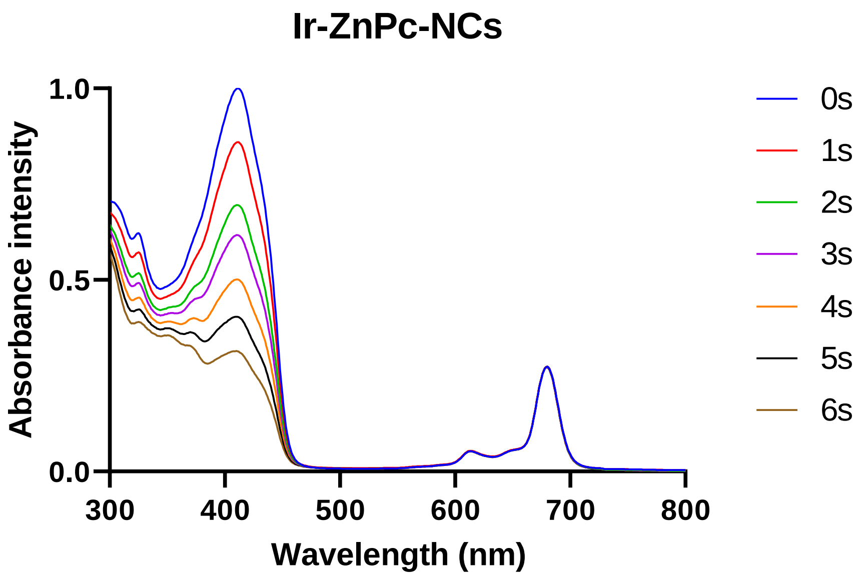

Figure S18. Changes in the absorption spectra of DPBF resulting from the irradiation with red LED light in the presence of **Ir-ZnPc** conjugate, either free (top) or encapsulated (bottom).

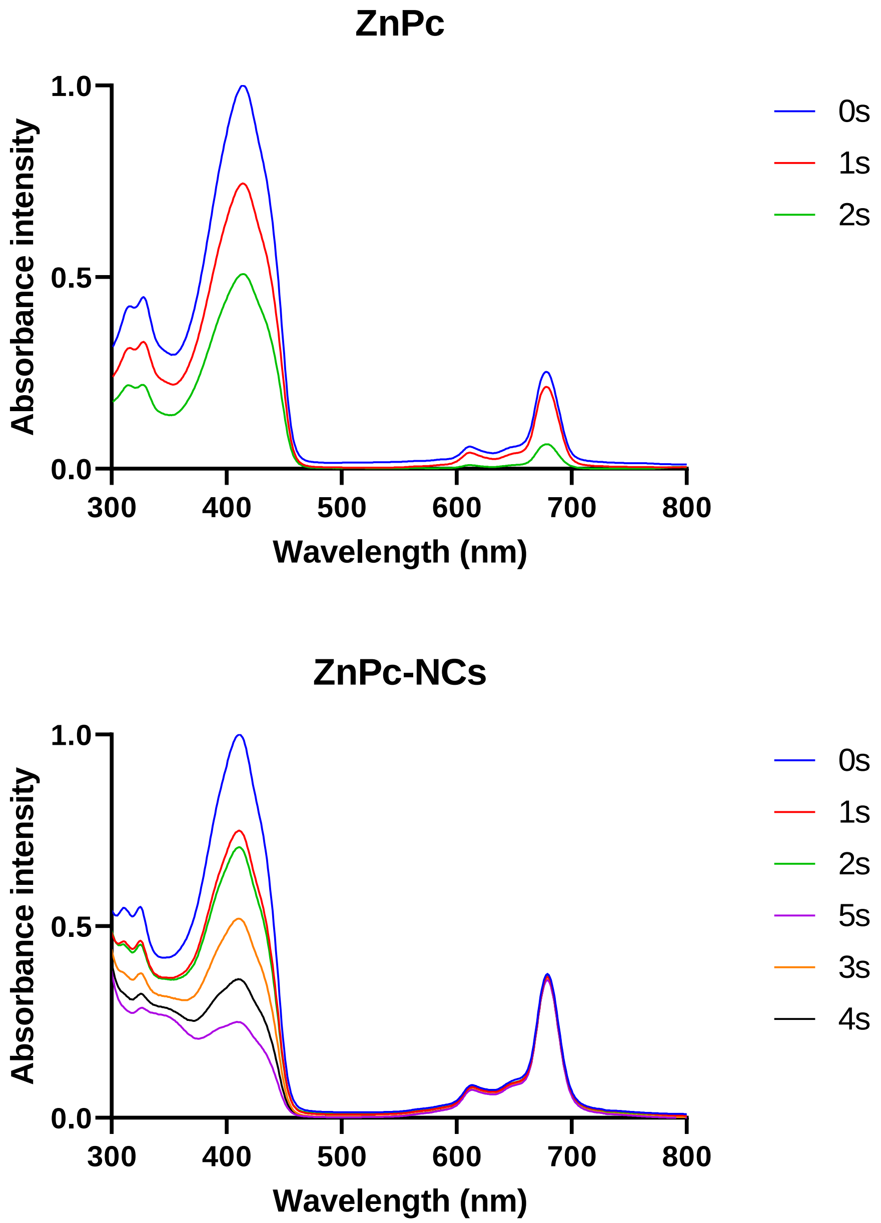

Figure S19. Changes in the absorption spectra of DPBF resulting from the irradiation with red LED light in the presence of **ZnPc**, either free (top) or encapsulated (bottom).

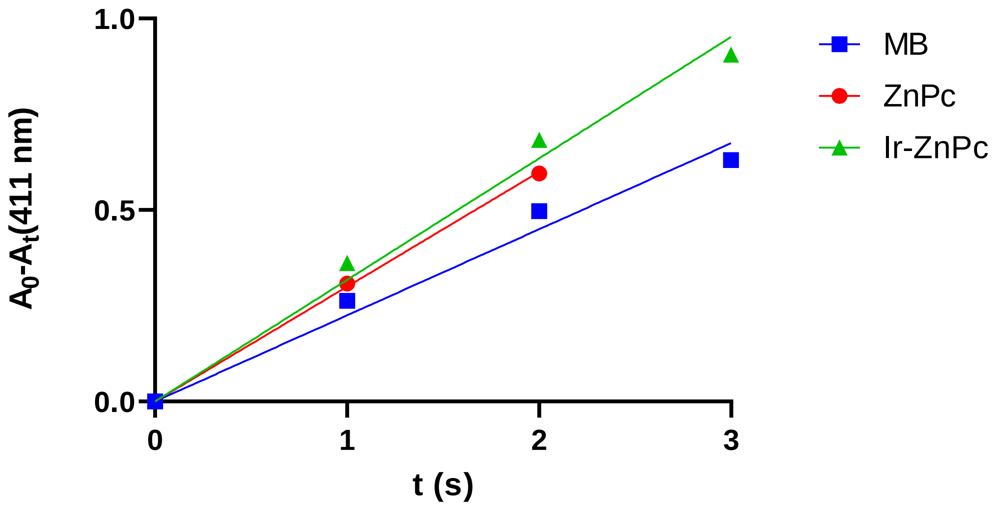

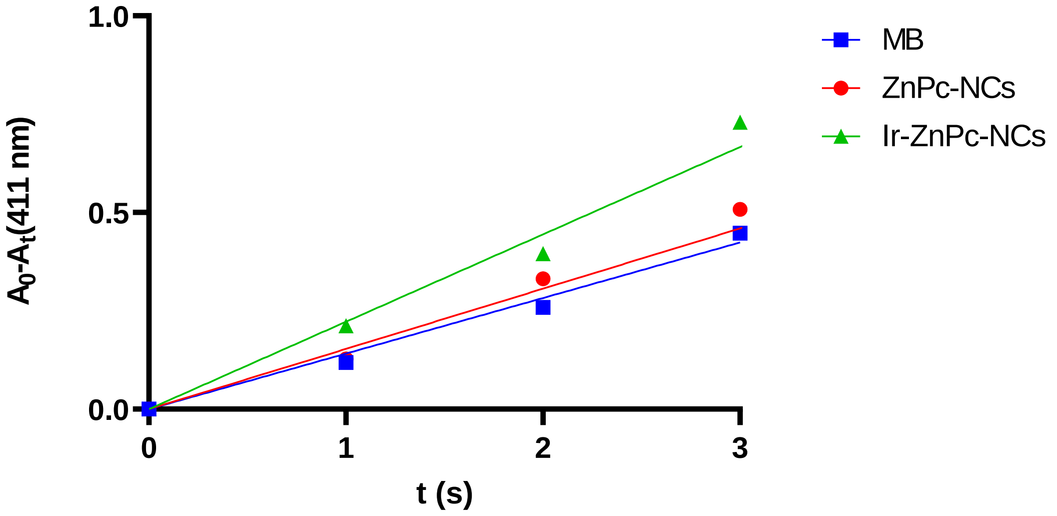

Figure S20. Plot of the changes in the absorbance (A_0-A_t) of DPBF at 411 nm against irradiation time in the presence of the standard sensitizer methylene blue and the investigated compounds, **ZnPc** and **Ir-ZnPc**, either free or encapsulated.

5.- Superoxide and hydroxyl radical measurements

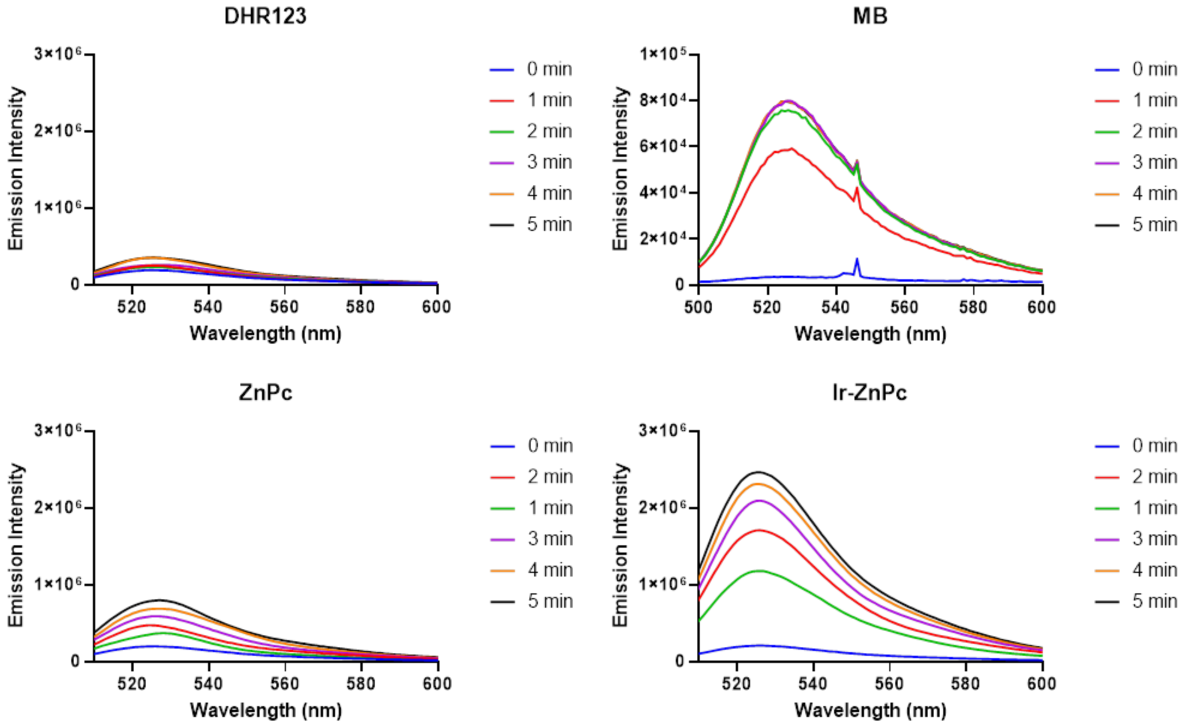

Figure S21. Photogeneration of superoxide by **ZnPc** and **Ir-ZnPc**. Increase of the fluorescence spectra emission of DHR123 upon photoirradiation of **ZnPc**, **Ir-ZnPc** and methylene blue (**MB**) or without any compound (DHR 123) at 620 nm in PBS (0.2 % DMSO). DHR123 was excited at 500 nm.

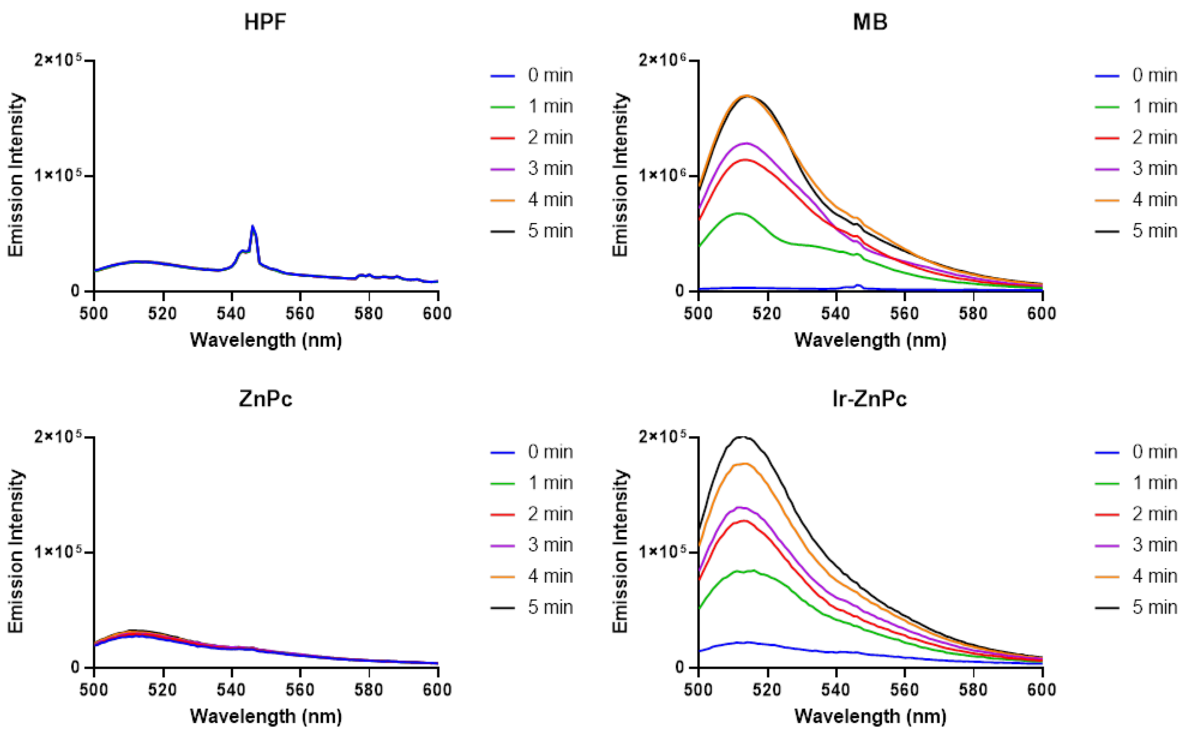

Figure S22. Photogeneration of superoxide by **ZnPc** and **Ir-ZnPc**. Increase of the fluorescence spectra emission of HPF upon photoirradiation of **ZnPc**, **Ir-ZnPc** and methylene blue (**MB**) or without any compound (HPF) at 620 nm in PBS (0.2 % DMSO). HPF was excited at 490 nm.

6. Confocal microscopy studies and lipophilicity

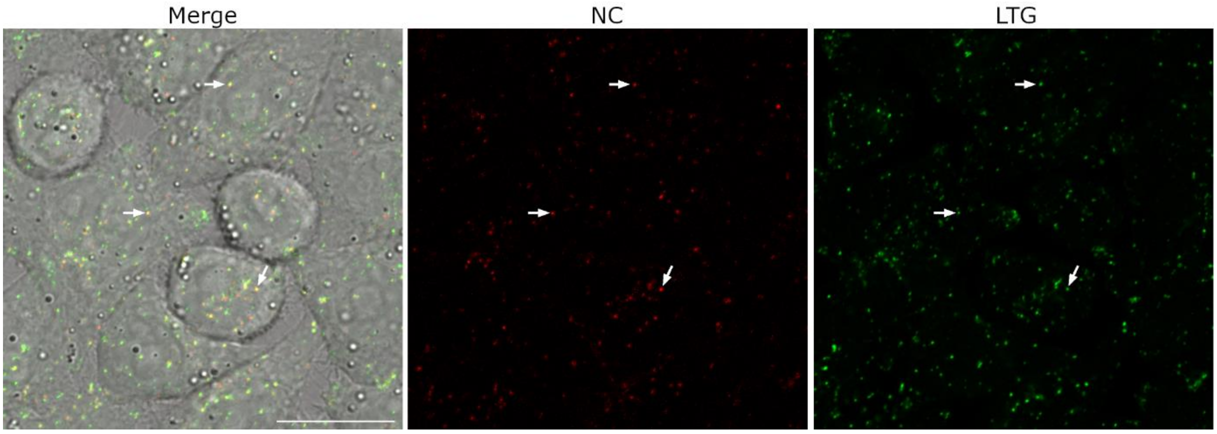

Figure S23. Colocalization studies with **ZnPc-NCs** and LysoTracker Green FM. Single confocal plane of HeLa cells incubated with **ZnPc-NCs** (10 μM, red) and LTG (0.2 μM, green). Left: merge of bright field, **ZnPc-NCs** and LTG staining; center: **ZnPc-NCs**' signal; right: LTG signal. White arrows point out **ZnPc-NCs**' vesicles colocalized with LysoTracker staining. Scale bar: 20 μm

Table S6. Pearson and Manders (M1 and M2) colocalization coefficients extracted from the analysis of **ZnPc-NCs** images under red-light excitation.

**Pearson**	0.519
**M1**	0.677
**M2**	0.295

Table S7. Log *P* values of **Ir**, **ZnPc** and **Ir-ZnPc** in octanol/water.

Compound	log *P*
**Ir**	+2.07
**ZnPc**	+3.57
**Ir-ZnPc**	+4.00

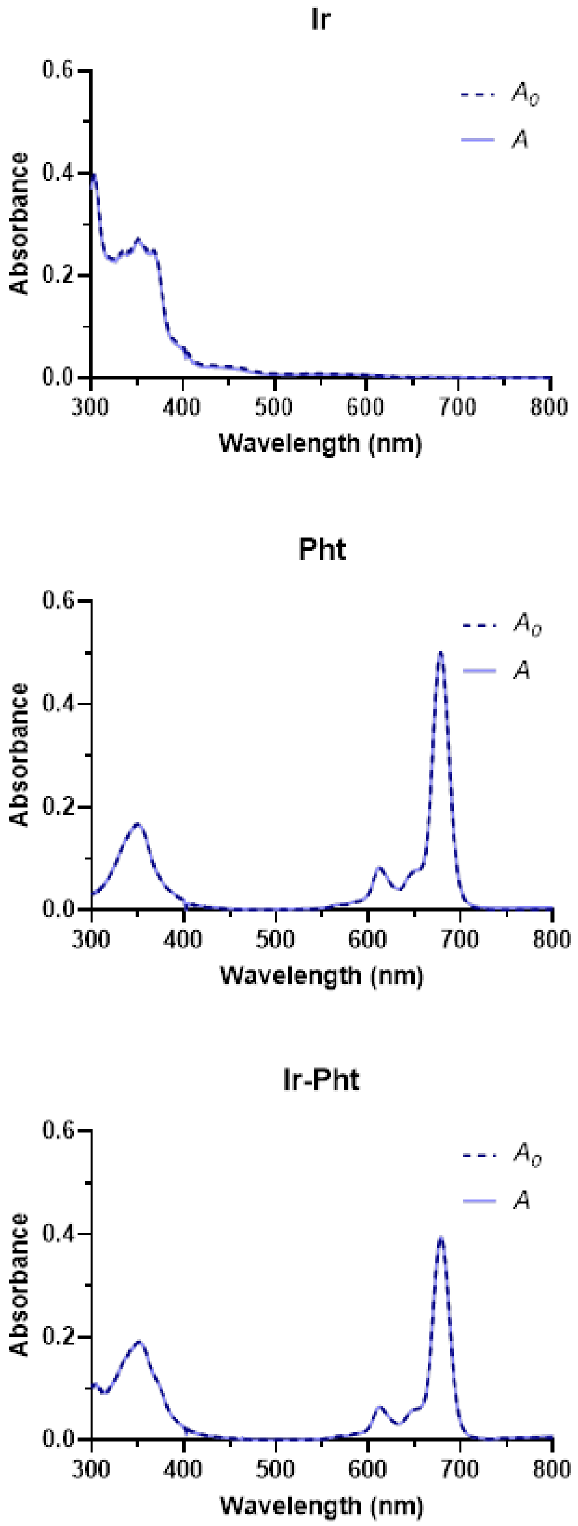

Figure S24. UV-Vis spectra of **Ir** (left), **ZnPc** (middle) and **Ir-ZnPc** (right) used for log *P* determination. The spectra of the reserved aliquots (A_0) are shown in dark blue dashed lines, whereas the spectra of the octanol phases of the O/W mixtures (*A*) are shown in light blue solid lines.

7. Photobiological studies

7.1. Phototoxicity evaluation

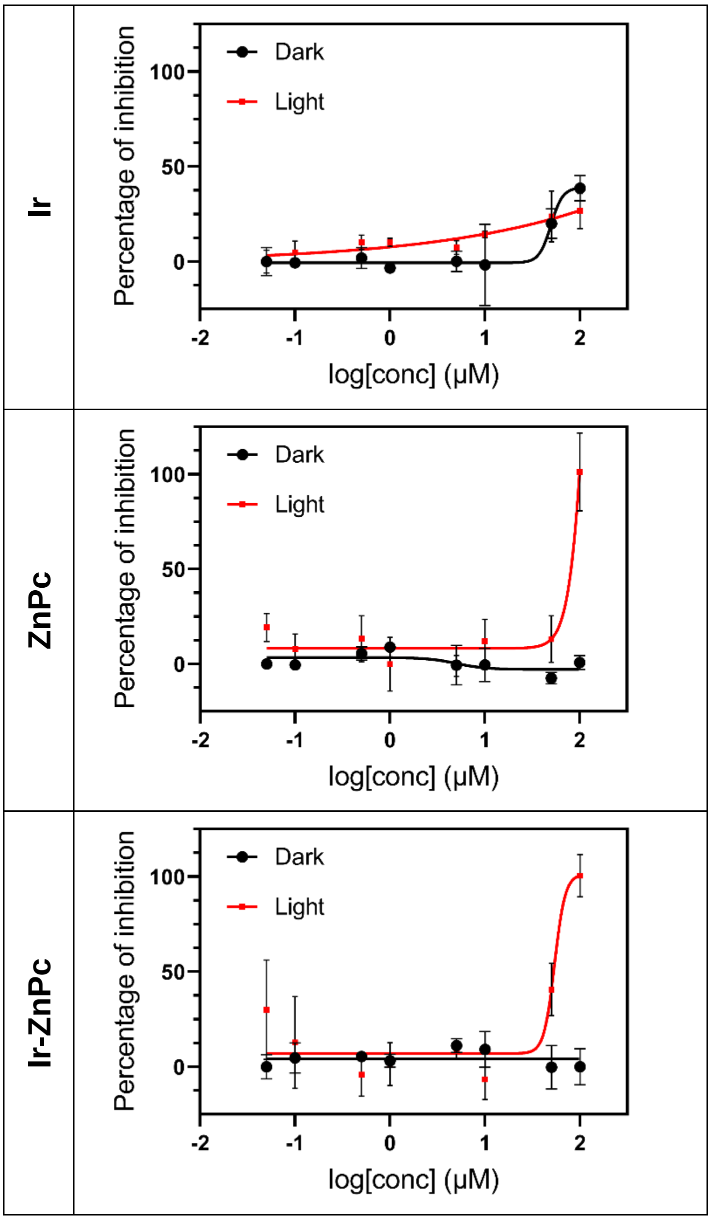

Figure S25. Dose-response curves of **Ir**, **ZnPc** and **Ir-ZnPc** in HeLa cells under dark or after red light irradiation under normoxia (21% O_2). Light irradiation condition: 630 nm, 1 h, 89 mW cm^{-2}.

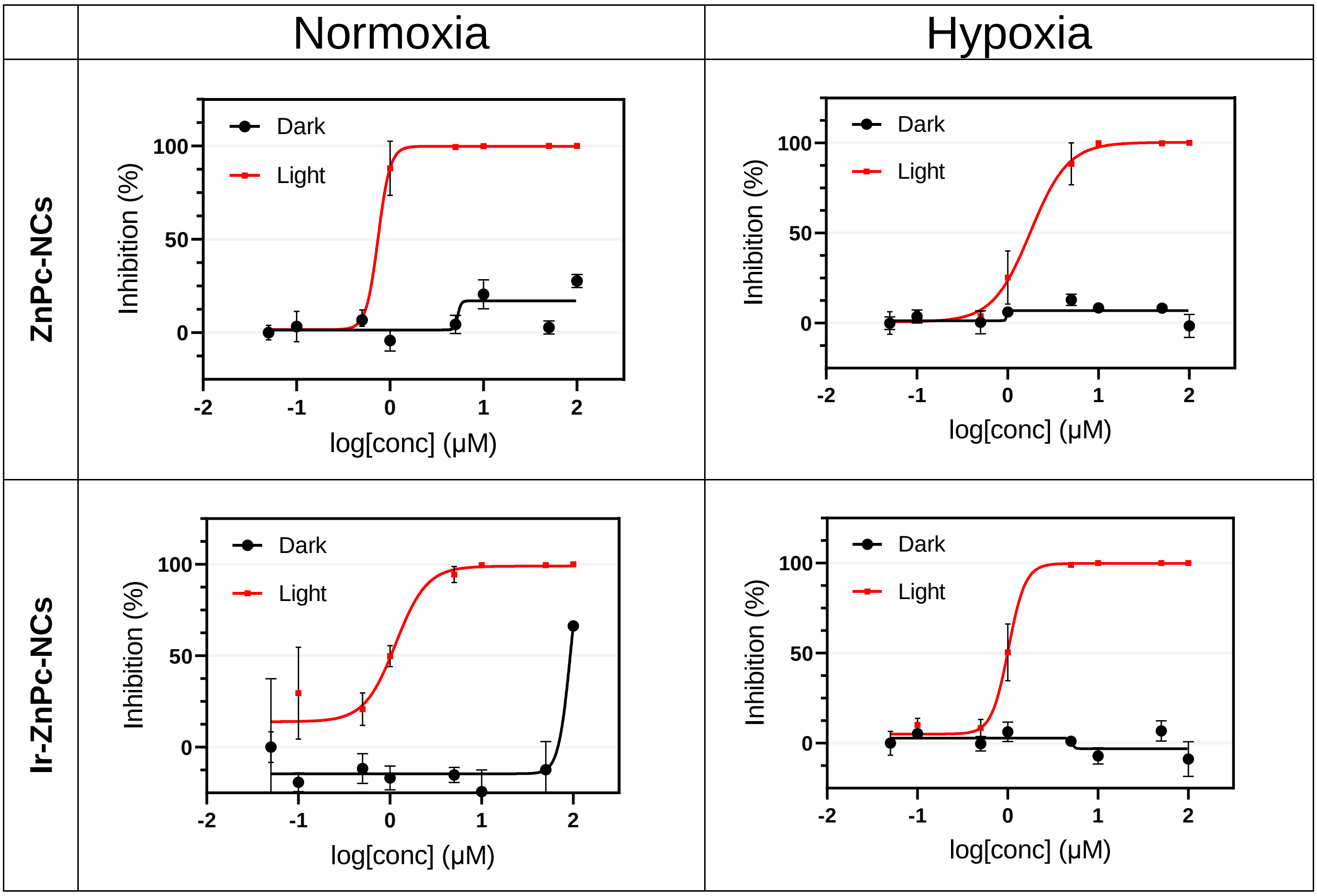

Figure S26. Dose-response curves for **ZnPc-NCs** and **Ir-ZnPc-NCs** in HeLa cells in the dark or after red light irradiation under normoxia (21% O_2) and hypoxia (2% O_2). Light irradiation condition: 630 nm, 1 h, 89 mW cm^{-2}.

7.2 Photogeneration of ROS

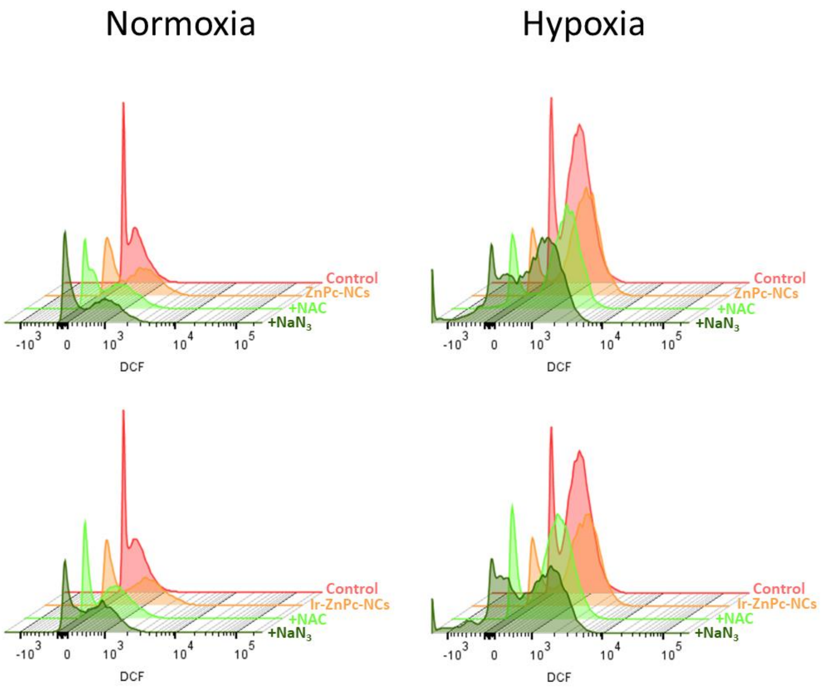

Figure S27. The offset histogram overlay of ROS production in Hela cells co-treated for 1 h with the encapsulated compounds (2.5 μM) and ROS scavengers NAC and NaN_3 followed by 1 h irradiation with red light.

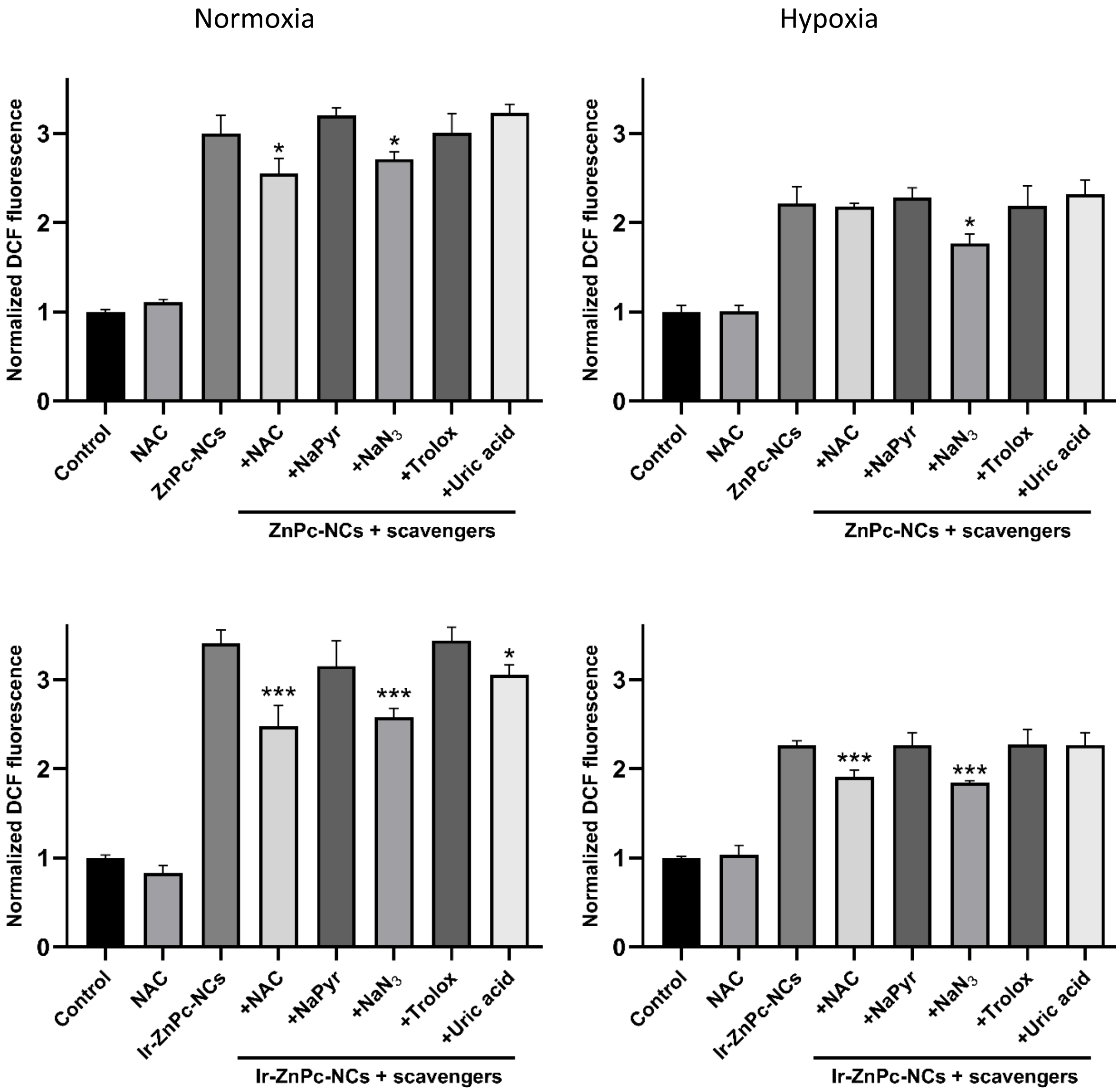

Figure S28. ROS levels in HeLa cells upon irradiation treatments with 2.5 μM of **ZnPc-NCs** and **Ir-ZnPc-NCs** (1 h in the dark, followed by 1 h red light irradiation) and then stained with DCFH-DA for 30 min at 37°C. Statistical significance indicated by *p < 0.05, **p < 0.01, and ***p < 0.001 using unpaired t-test. Data represented as mean ± SD (n = 2 replicates).

7.3 Assessment of photocytotoxicity in 3D multicellular spheroids

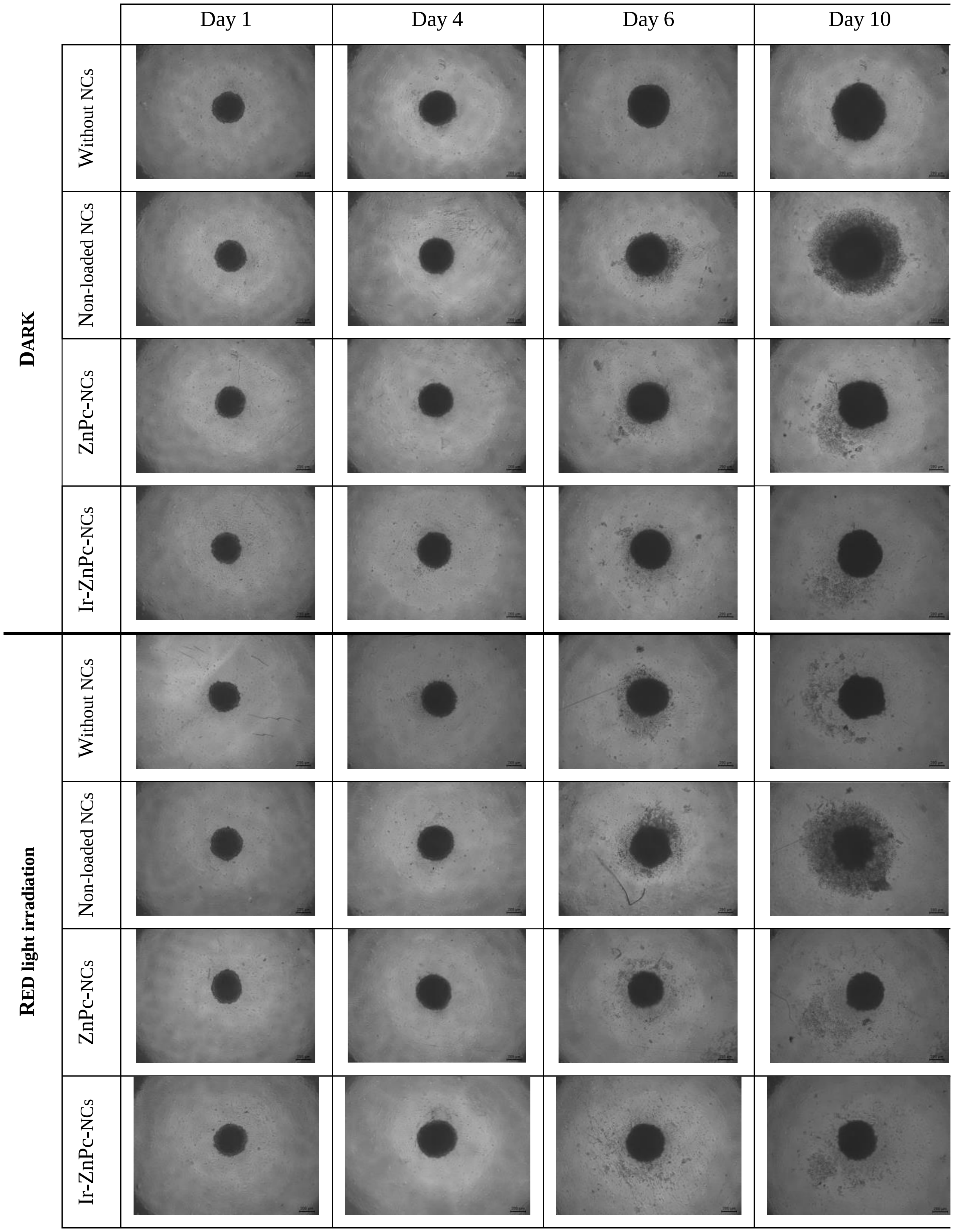

Figure S29. Alterations in MCTS relative diameter and volume observed over 10 days after treatment with 50 μM of **ZnPc-NCs** and **Ir-ZnPc-NCs**, followed by 1-hour red light irradiation. The control was kept in dark conditions. Scale bar: 200 μm.
